# Significant impact of *Bacillus licheniformis* DW4, *Salinicoccus sesuvii* DW5 and *Paenalcaligenes suwonensis* DW7, in mitigation of seawater stress on the growth and productivity of *Vicia Faba* cultivated in Qalabshu semi-field soil

**DOI:** 10.1186/s12870-025-08055-8

**Published:** 2026-02-04

**Authors:** Dalia Wael, Yasser El-Amier, WesamEldin I. A. Saber, Ashraf Elsayed

**Affiliations:** 1 Botany Department, Faculty of Science, Mansoura University, Mansoura, 35516 Egypt; 2 Microbial Activity Unit, Microbiology Department, Soils, Water and Environment Research Institute, Agricultural Research Center, Giza, 12619 Egypt

**Keywords:** Bacteria, Salinity stress, Seawater, Newly-reclaimed soil, Vicia faba, Seed priming.

## Abstract

**Background:**

Salinity severely limits sensitive crops like *Vicia faba* in newly-reclaimed lands. Costly mitigation is unsustainable; Plant Growth-Promoting Bacteria (PGPB) from halophytes provide a cheap, effective, eco-friendly solution to alleviate salt stress.

**Objective:**

To address Egypt’s food security and overpopulation challenges, this study aimed to enhance *Vicia faba* growth and yield in newly-reclaimed soils using PGPB. A key goal was to conserve freshwater by testing the feasibility of cultivating faba beans with saltwater and PGPB.

**Methods:**

Single and a mixture treatment of *Paenalcaligenes suwonensis* DW7 OR147937.1, *Salinicoccus sesuvii* DW5 OR083408.1 and *Bacillus licheniformis* DW4 OR083404.1 were utilized in *Vicia faba* seeds priming in semi-field and irrigated with different concentrations of seawater. Measurements encompassed *Vicia faba*’s key growth metrics, the extent of nodule formation, detailed root anatomy, concentrations of photosynthetic pigments and antioxidants, evaluations of soil fertility, comprehensive yield parameters, and the total protein content found in its seeds.

**Results:**

Treatments *P. suwonensis* DW7 and B mix (*B. licheniformis* DW4 + *S. sesuvii* DW5 + *P. suwonensis* DW7) consistently resulted in optimal performance across growth, physiological, and yield metrics. The improved salt tolerance stemmed from enhanced antioxidant activity, proline synthesis, and significant root anatomical changes (e.g., enlarged vascular cylinders). Critically, under maximal stress (150 mM NaCl), B mix-treated plants achieved a 40% yield increase compared to control plants grown in freshwater, while failing to produce seeds under salt stress.

**Conclusion:**

This study demonstrates that the treatment *P. suwonensis* DW7 and the B mix, significantly enhances the yield and growth of *Vicia faba* within salty soil. This innovation offers a promising solution for bolstering enhanced safety of food by making it possible for salt-susceptible crops to thrive but a broader range of agricultural produce in newly-reclaimed soils. Ultimately, this approach is poised to play a crucial role in mitigating Egypt’s impending agricultural crisis.

**Graphical abstract:**

Significant impact of*Bacillus licheniformis* DW4, *Salinicoccus sesuvii* DW5 and *Paenalcaligenes suwonensis* DW7 isolated from *Suaeda pruinosa* Lange and *Arthrocnemum macrostachyum* (Moric.) K.koch, in mitigation of seawater stress on the growth and productivity of *Vicia faba* cultivated in Qalabshu semi-field soil.

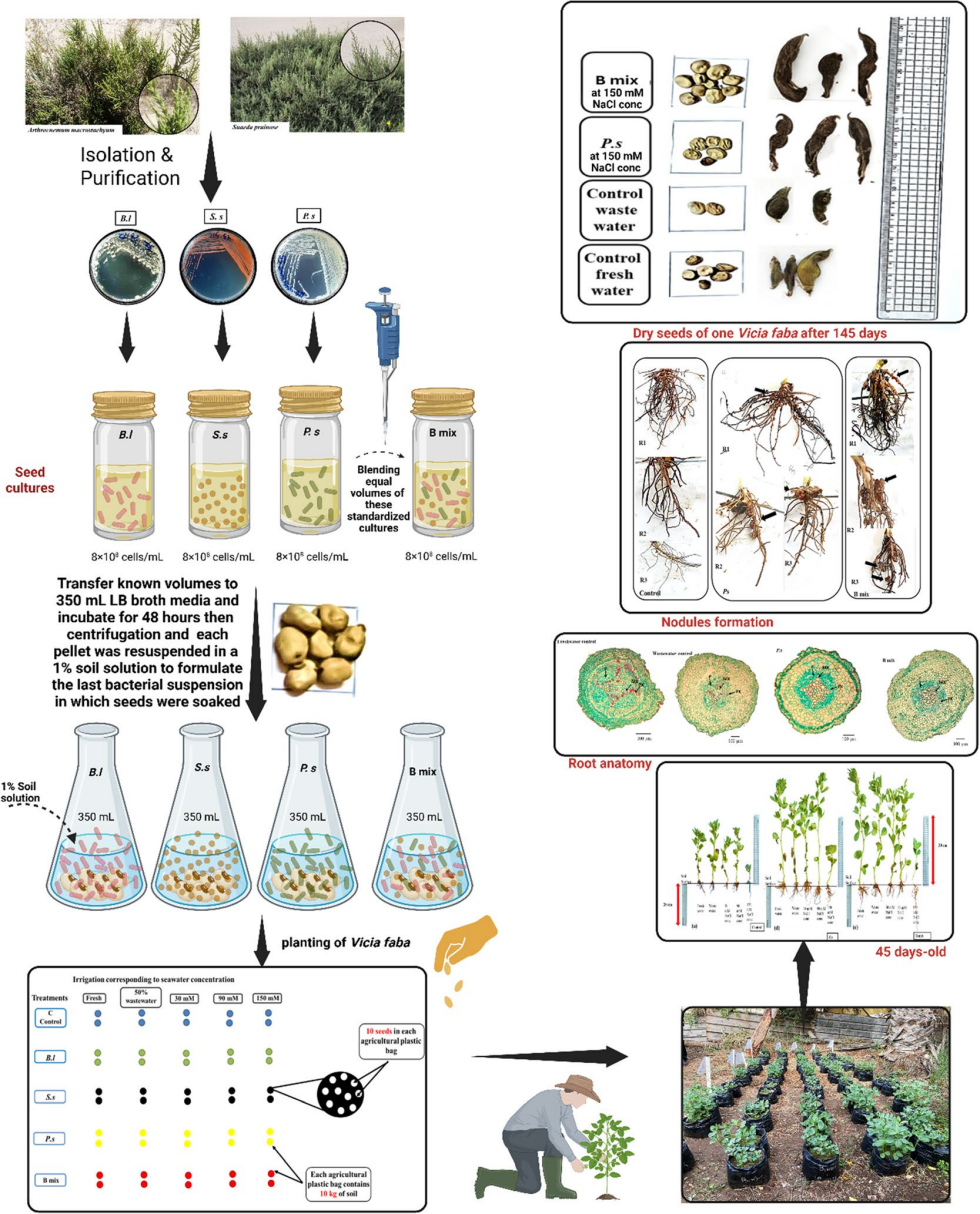

**Supplementary Information:**

The online version contains supplementary material available at 10.1186/s12870-025-08055-8.

## Introduction

Approximately 50% of fertile soil is affected by salinity and gradual salinization [1, 2]. Newly-reclaimed soils face several issues including high salt content and low soil organic carbon (SOC) stocks or low soil fertility and limited water resources damaging crop yield for food and feed [3]. Lower SOC levels are associated with fewer microorganisms and consequently reduced soil fertility [4].

*Vicia faba* is a strategic leguminous crop in Egypt, serving as a vital vegetarian protein source [5, 6], yet approximately 60% of national demand is met by imports [7]. The inherent susceptibility of the faba bean to salinity is the primary factor limiting its successful cultivation in newly-reclaimed soils. The detrimental effects of salt stress are initiated by the creation of osmotic pressure, diminishing water availability and restricting root uptake; this is immediately followed by ion imbalance and cellular toxicity as excessive salts accumulate [8]. In order to fulfill the growing need for food brought on by population growth, the country is exploring alternative water resources like seawater for expanding irrigated agriculture and utilizing newly-reclaimed soil [9, 10].

Salinity is a primary constraint on global agriculture. Its detrimental effects on plant health include compromised photosynthetic capacity (reduced chlorophyll), diminished light harvesting area, and restricted gas exchange (decreased stomatal conductance) [11, 12] and encourages reactive oxygen species (ROS) generation that damages vital biological constituents, such as proteins, lipids, and DNA [13]. This damage impairs enzyme function and can even lead to the breakdown of the membrane of the cell, disrupting the protective barrier of the cell [14].

Salinity severely impacts crops like faba beans by creating a multi-faceted stress response. High salt concentrations lead to osmotic stress, restricting water uptake, and ionic stress, as toxic ions like sodium and chloride accumulate [15]. This buildup disrupts essential plant functions [16], causes nutrient deficiencies (e.g., in phosphate [17], calcium, potassium, iron, and zinc [18]), which eventually results in oxidative stress [19, 20]. These combined effects inhibit photosynthesis, reduce nitrogen uptake, impair growth, and can cause plant death, severely limiting agricultural productivity [21].

Salinity disrupts a plant’s hormonal balance. Salt stress triggers a fundamental hormonal imbalance: stress hormones (such as abscisic acid and ethylene) are upregulated, concurrent with a decline in levels of growth-promoting hormones (specifically indole-3-acetic acid (IAA) and gibberellic acid (GA-3). This imbalance of phytohormones significantly reduces overall plant growth, including the development of shoots and roots [22–24]. Given the critical role of the root system in water and nutrient uptake, crop productivity is directly impacted by salt stress, particularly sodium chloride, which affects root anatomy and morphology [25–28]. Salt stress influences plants’ photosynthetic rates to drop because it reduces photosynthetic pigments like chlorophyll a and b and carotenoids and partial stomatal closure. Furthermore, the cellular salt overload generates ROS. This oxidative stress damages the photosynthetic machinery and initiates a cascade of degradation, leading to the destruction of chlorophyll and structural harm to the chloroplasts [29, 30]. Under stress conditions, the rate of ROS production exceeds the capacity of the plant’s antioxidant system such as catalases, peroxidases, polyphenol oxidase, phenylalanine ammonia lyase, total phenolic and flavonoids often leading to oxidative stress to neutralize them, frequently resulting in oxidative stress. Photosynthetic pigments are oxidatively damaged when cellular ROS levels rise sharply, interfering with regular metabolic functions [30, 31].

Because salinity stress weakens antioxidant defenses and increases the creation of harmful ROS, it speeds up nodule senescence, leading to a decline in nitrogen-fixing activity [32]. In sensitive, protein-rich crops like the faba bean, salt stress negatively impacts nutritional quality by decreasing total seed protein levels [33] This decline is hypothesized to result from either the suppression of protein synthesis pathways or a corresponding increase in proteolytic activity and catabolic degradation processes.

The capacity of plant extracts from leaves and seeds to alleviate salt stress is attributed to their complex profile of active compounds, notably proline and a range of enzymatic and non-enzymatic antioxidant defenses [34]. One of the most common and effective metabolic adjustments in stressed plants is the accumulation of key osmolytes. As a critical component of the stress response, the metabolic accumulation of proline is frequently observed under conditions of water deficit and salinity. Proline serves a multifaceted protective role, functioning both as a compatible osmoprotectant [35] and a crucial agent in scavenging harmful ROS [36]. This detoxification role is supported by the plant’s comprehensive enzymatic and non-enzymatic antioxidant system, which collectively protects against the severe oxidative damage caused by free radicals generated under salt stress. They achieve this by directly neutralizing free radicals, binding harmful metals, and inhibiting oxygen-based reactions. Faba beans are a promising legume crop known for their rich antioxidant content [37]. Antioxidants can originate from various sources. These include enzymatic systems like superoxide dismutase, catalase, and glutathione peroxidase; non-enzymatic proteins such as ferritin and albumin; and low molecular weight compounds like phenolic compounds, flavonoids, glutathione, and vitamins A, C, and E [34, 38–40]. Environmental stress causes a reduction of the balance between reactive oxygen species and antioxidant defenses of plants [41].

Salinity severely limits legume productivity by negatively affecting host plant growth, symbiotic development, and the nitrogen-fixing capacity of root nodule bacteria [42]. The most frequent limiting soil nutrient that lowers agricultural production is nitrogen (N). In root nodules, Rhizobium transfers fixed nitrogen (NH_3_) into the cytoplasm of the host plant, where it is further converted into amino acids. In agriculture under salt stress, PGPB enhances the efficiency and success of the legume-*Rhizobium* symbiosis, ensuring a stable and renewable source of fixed nitrogen necessary for plant growth and sustained nodule function [43–45].

In all plant tissues—roots, seeds, stems, and leaves—endophytic bacteria coexist peacefully and without causing harm [46, 47]. Through the production of numerous kinds of phytohormones including gibberellin (GA) and IAA [48, 49] and they foster plant growth. They are believed to be a significant source of compounds that help plants tolerate salt, natural antibacterial agents, and secondary metabolites [50]. Additionally, they generate osmolytes like proline [51] and secondary substances such exopolysaccharides [52]. Critically, these compounds, when subjected to salt stress, regulate the plant’s defense mechanism and trigger the antioxidant enzymes, providing the physiological foundation for survival and productivity under saline irrigation [53].

Earlier studies have demonstrated the plant–microbe interaction and the beneficial role of microbes in enhancing plant stress tolerance and improving crop development. Among the diverse communities of PGPB, the Gram-positive, spore-forming genus *Bacillus* stands out and is increasingly preferred. This is due to its inherent stability and exceptionally long shelf life, a direct benefit of its spore-forming capacity. Furthermore, various *Bacillus* species demonstrate remarkable versatility in supporting crop health and exhibit high biochemical adaptability, particularly under environmental stresses like saline or seawater stress, making them highly effective in challenging agricultural settings [54]. For example, *Bacillus subtilus* from wild *Phragmites australis* and *Arthrochemum macrostachyum*, has enhanced the growth and yield of both maize and faba beans [55, 56]. Additionally, *Bacillus cereus*, found on *Stevia rebaudiana* leaves, promotes growth and acts as a biocontrol agent [57]. These examples highlight the successful application of robust PGPB in saline environments, justifying their selection as candidates for our study utilizing seawater irrigation.

Other PGPB strains, including *Serratia plymuthica* [58], *Pseudomonas yamanorum*, *Rahnella aquatilis*, *Pseudomonas fluorescens* [59], and *Enterobacter hormaechei* [60], isolated from soil and plant rhizospheres, have also successfully promoted faba bean growth. The ability of bacteria to adhere and colonize surfaces drives their shift from a free-swimming to a static existence. This transition is defined by the formation of biofilms, which are vital mediators of plant-microbial interaction in agriculture [61].

Therefore, this study was designed to systematically assess the efficacy of novel halophyte-derived PGPB (single and mixed consortia) in mitigating salt stress and enabling the successful cultivation of salt-sensitive *Vicia faba* in highly saline, newly-reclaimed soils under semi-field conditions with seawater irrigation. We specifically investigated the resultant improvements in growth, yield, and nutritional value, while detailing the underlying protective mechanisms, including changes in root anatomy, photosynthetic pigments, and the activation of antioxidant defenses. This methodology aims to validate a viable, sustainable approach to unlock agricultural productivity in newly-reclaimed soils, thereby enhancing Egypt’s food security and helping address its looming agricultural crisis.

## Materials and methods

### Bacterial isolates, seeds and soil

*B. licheniformis* DW4, *S. sesuvii* DW5 and *P. suwonensis* DW7 were earlier isolated *from Suaeda pruinosa* Lange and *Arthrocnemum macrostachyum* (Moric.) K.koch. were successfully identified through 16 S rRNA gene sequencing, with the resulting sequence data archived in a gene bank, as earlier explained [62]. Every isolate can meet PGP (Plant Growth Promoting) criteria such as IAA, GA-3, siderophores, ammonia, hydrogen cyanide, exopolysaccharides, and has enzymatic activity by generating the enzymes cellulase, protease, catalase, amylase, and lipase. In addition to fixing atmospheric nitrogen and solubilizing phosphate, the strain is able to tolerate salinity levels reaching 3400 mM NaCl [62].

**Fig. 1:**
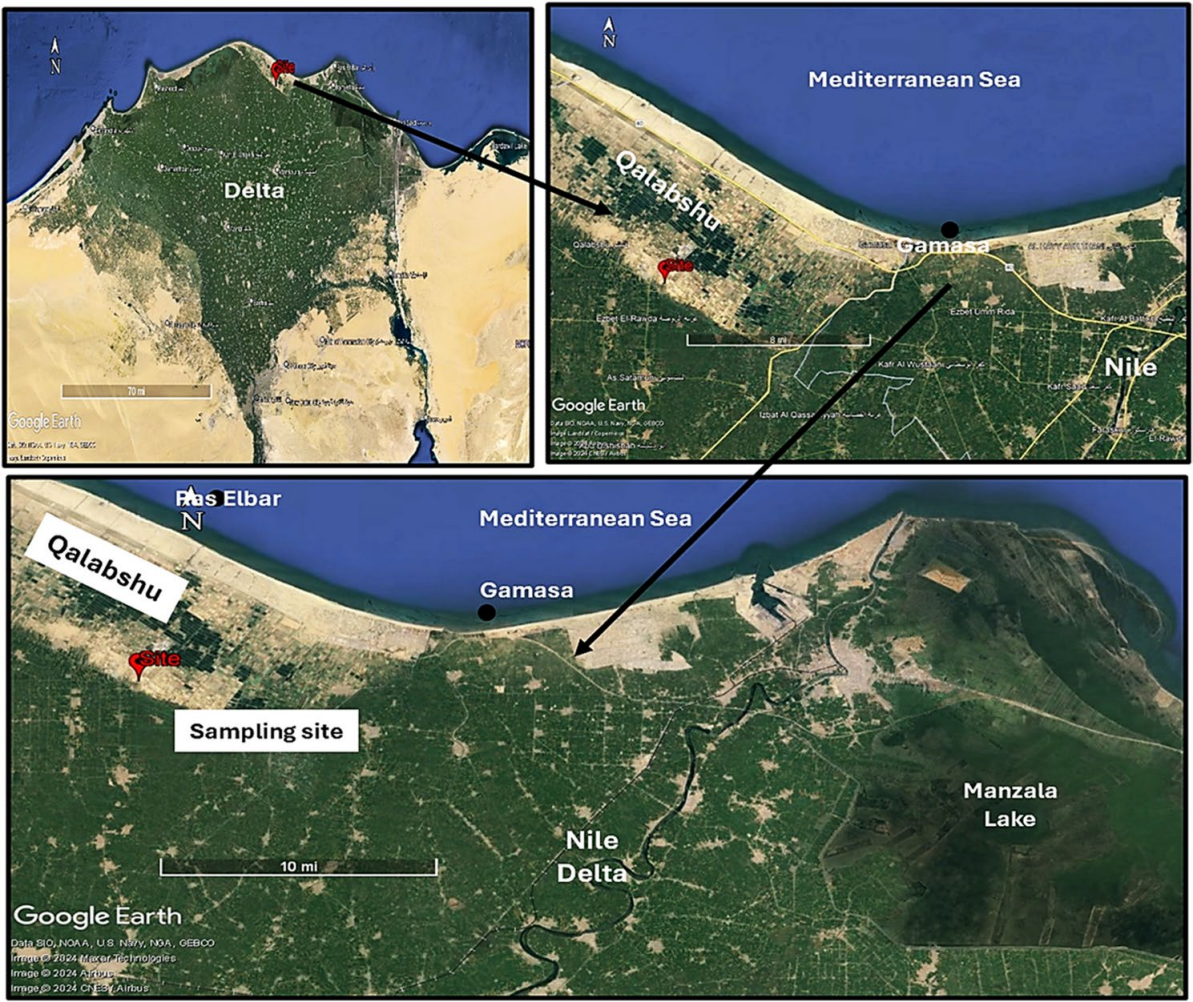
Map showing the site of samples (soil) collection from Qalabshu. (Google Earth Pro version; 7.3, https://earth.google.com/web/@-0.21047219,-4.7998464, 5072. 242 36397a ,19499383.57429804d,35y, 0h, 0t,0r/ data=OgMKATA)

Seeds of healthy *Vicia faba* L Mariout-2 were obtained from the Field Crop Research Institute (FCRI), Agricultural Research Centre, Giza, Egypt. To simulate saline conditions, newly-reclaimed, salt-affected soil and agricultural wastewater were collected from Qalabshu, located along the Mediterranean Deltaic coast in the Dakahlia and Damietta Governorates. The GPS coordinates for the collection site are 31°24’28.4” N, 31°22’30.7” E (Fig. 1).

### Agricultural wastewater analysis (S1)

Agricultural wastewater was analyzed in the Soil, Water & Environment Research Institute, Agricultural Research Centre, Giza, Egypt. The physical properties measured were pH and electrical conductivity (EC), reported in both deciSiemens per meter (dS/m) and parts per million (ppm). A Mettler Toledo pH and conductivity meter was used for these measurements, while chemical analysis of wastewater includes anions like; CO_3_^− 2^, HCO _3_^−,^ Cl^−^ and SO₄²^−^ were specifically measured using silver nitrate (AgNO_3_) and potassium chromate (K_2_​CrO_4_​) as indicators, cations like; Ca^2+^, Mg^2+^, Na^+^ and K^+^ exchangeable cations were determined utilizing ammonium acetate at a pH of 7 residue, ammonium (NH_4_^+^​), nitrate (NO_3_^−^​), boron (B), copper (Cu), iron (Fe), manganese (Mn), phosphorus (P), and zinc (Zn) were determined through extraction using diethylene-triamine-penta-acetic acid [63]. Finally, phosphorus, potassium, calcium, and sodium were analyzed using an inductively coupled plasma spectrophotometer after initial extraction [64]. Almost the results were higher than what is allowed in the irrigation of plants. Also, EC, anions, cations, Cl^−^, Na^+^, adsorbed sodium %, NH_4_^+^ and NO_3_ concentrations were extremely high.

### Bacterial inoculum preparation [65]

Individual strains of *B. licheniformis*, *S. sesuvii*, and *P. suwonensis* were initially cultured in LB broth media for 48 h as a preparatory step for the experiments. These cultures were standardized to a concentration of approximately 8 × 10^8^ cells/mL. A combined bacterial mixture B mix (*B. licheniformis* DW4 *+ S. sesuvii* DW5 *+ P. suwonensis* DW7) was created by blending equal volumes of these standardized cultures. For the final inoculum, this mixture was added to fresh LB broth and incubated again for 48 h. The bacterial cells were then pelleted via centrifugation, and the pellet was resuspended in a 1% soil solution to formulate the last bacterial suspension for the study.

### Semi-field biopriming and planting of *Vicia Faba*

**Fig. 2:**
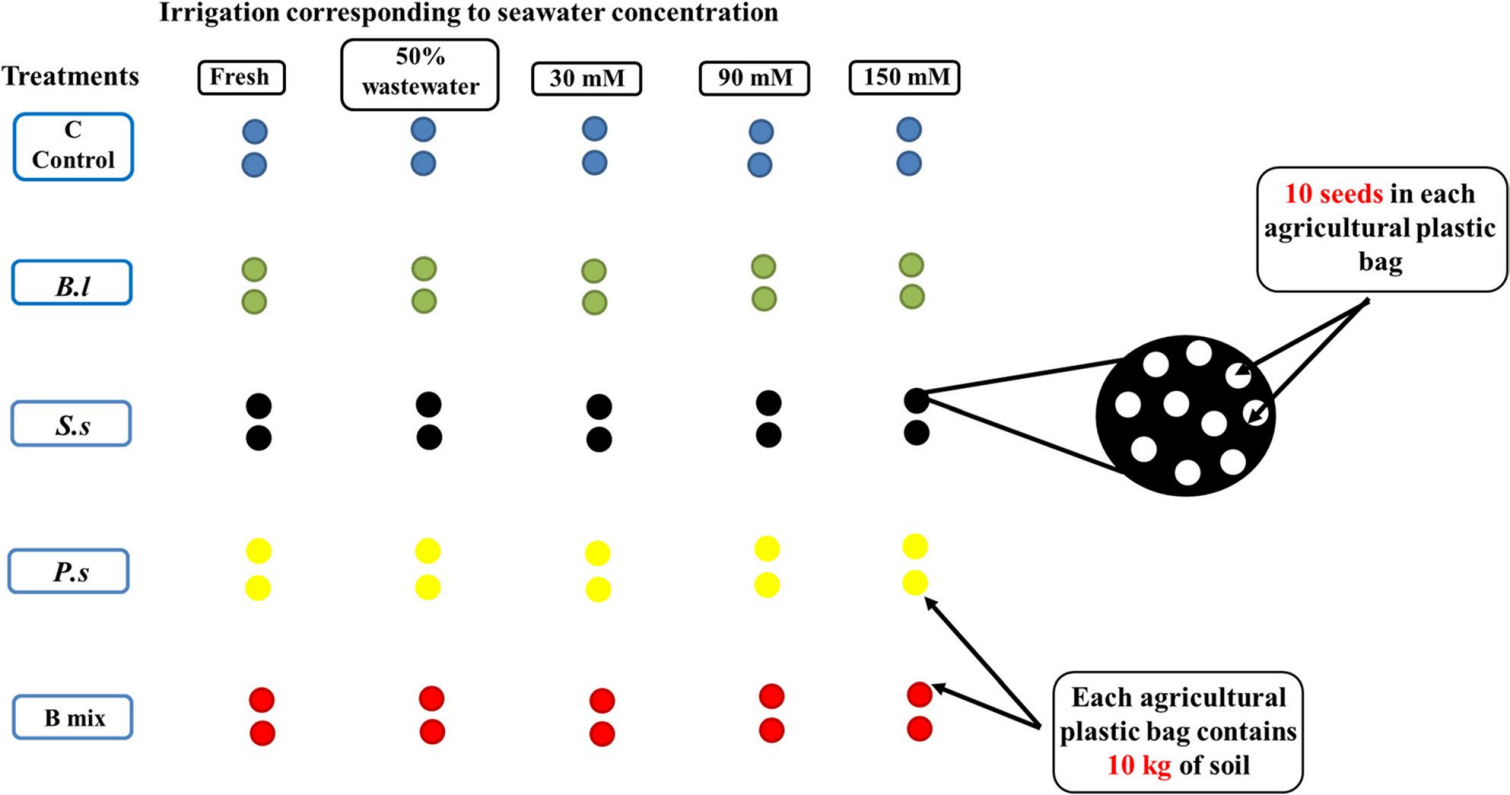
Planting map of *Vicia faba* after 145 days in semi-field conducted by PowerPoint. *Bl*: *B.licheniformis* DW4, *Ss*: *S. sesuvii* DW5, *Ps*: *P. suwonensis* DW7 and B mix: *(B. licheniformis* DW4* + S. sesuvii* DW5* + P. suwonensis* DW7)

An on-site, single semi-field trial utilizing a completely randomized design was conducted at the Agriculture Faculty, Mansoura University, Egypt, on November 15, 2023. The study aimed to test whether PGPB could enhance the growth of the salt-sensitive faba bean variety with EC threshold of 2.0 dS/m [66], *Vicia faba* L. Mariout-2, when grown in saline soil environments. Seeds were surface-sterilized as previously mentioned [57, 65] and then primed (soaked for 24 h) in different bacterial suspensions, each set to a certain optical density (OD 600 nm​) of 0.8. Control seeds were primed in either fresh Nile water or agricultural wastewater. After priming, replicas of twenty primed seeds were planted in 10-kg bags (bag size 0.025 m^3^) of saline soil collected from a newly reclaimed area with EC of 5.48 dS/m and classified as loamy sand soil and composed of sand (75.5%), silt (22%), and clay (2.5%). Each treatment was performed in duplicate (Fig. 2 ). No additional nitrogen-fixing bacteria were added to the soil.

All agricultural plastic bags containing primed seeds were left in the semi-field with plot size of 6 × 3 m^2^ and irrigated with Nile water, agricultural wastewater 50% (1 freshwater: 1wastewater (v/v)), and gradually with 30 mM NaCl concentration (5% seawater),90 mM NaCl concentration (15% seawater) and 150 mM NaCl concentration (25% seawater) for 145 days samples according to the gradual watering system as follow:

1. *Freshwater Control*: Plants were continuously irrigated with freshwater throughout the entire growth period.
2. *50% Agricultural Wastewater Control*: Plants were continuously irrigated with a 50% dilution of agricultural wastewater throughout the entire growth period.
3. *30 mM NaCl*: Plants were irrigated with Freshwater for the first month, followed by continuous irrigation with 30 mM NaCl until harvest.
4. *90 mM NaCl (Progressive Stress)*: Plants were subjected to a progressive increase in salinity:
  *Month 1*: Freshwater irrigation.
  *Following 2 Weeks*: 30 mM NaCl irrigation.
  *Thereafter*: Continuous irrigation with 90 mM NaCl until harvest.
5. *150 mM NaCl (Maximal Progressive Stress)*: Plants received the highest concentration of salt using a step-wise method to ensure establishment:
  *Month 1*: Freshwater irrigation.
  *Following 2 Weeks*: 30 mM NaCl irrigation.
  *Next 2 Weeks*: 90 mM NaCl irrigation.
  *Thereafter*: Continuous irrigation with 150 mM NaCl until harvest.

Samples of plants and soil were gathered throughout three key phases of growth: vegetative (45 days), flowering (110 days), and fruiting (145 days). Various parameters of growth were then measured, along with other relevant plant and soil criteria.

### Germination percentage and plant survival assessments

Every day, measurements were made of seed germination and damping-off. The resulting Germination Percentage (G%) was calculated using the formula:

1 $$\begin{aligned}\:\mathbf{G}\mathbf{e}&\mathbf{r}\mathbf{m}\mathbf{i}\mathbf{n}\mathbf{a}\mathbf{t}\mathbf{i}\mathbf{o}\mathbf{n}\:\mathbf{p}\mathbf{e}\mathbf{r}\mathbf{c}\mathbf{e}\mathbf{n}\mathbf{t}\mathbf{a}\mathbf{g}\mathbf{e}\:\left(\mathbf{G}\mathbf{\%}\right)\\&=\frac{\mathbf{N}\mathbf{u}\mathbf{m}\mathbf{b}\mathbf{e}\mathbf{r}\:\mathbf{o}\mathbf{f}\:\mathbf{g}\mathbf{e}\mathbf{r}\mathbf{m}\mathbf{i}\mathbf{n}\mathbf{a}\mathbf{t}\mathbf{e}\mathbf{d}\:\mathbf{s}\mathbf{e}\mathbf{e}\mathbf{d}\:\:}{\mathbf{T}\mathbf{o}\mathbf{t}\mathbf{a}\mathbf{l}\:\mathbf{n}\mathbf{u}\mathbf{m}\mathbf{b}\mathbf{e}\mathbf{r}\:\mathbf{o}\mathbf{f}\:\mathbf{s}\mathbf{e}\mathbf{e}\mathbf{d}}\times\:100\:\:\end{aligned}$$

The percentage of dead seeds before they emerged was used to assess pre-emergence damping-off, and the damage to emerged seedlings was used to assess post-emergence damping-off.

### Parameters of growth measurements

45 and 110-day-old faba bean plants were sampled to assess parameters of vegetative growth. For each stage, five plants were measured for height, leaves and branches number, fresh and dry weight of both roots and shoots and root and shoot length [67].

### Estimation of nodules formation

In the semi-field experiment, nodule formation was assessed at two critical developmental stages: 45 days and 110 days post-planting. At each point, the number and fresh weight of nodules were quantified for three replicate plants per treatment.

### Transverse section in *Vicia Faba* root

The anatomy of *Vicia faba* roots was performed in accordance with the technique of Feder N, et al. [68] and Yang J-Y, et al. [69]. Root segments (0.5 mm thick) were collected from fresh root samples materials of roots of *Vicia faba* were harvested at 110 days after growth and washed with water, then cut and immediately fixed in formalin-acetic acid-alcohol (10:5:85v\v). The fixed samples were then completely dehydrated in tertiary butyl alcohol, immersed in paraffin, cut into 10–15 μm sections using a rotary microtome instrument, and stained with fast green and safranin and photographed under a microscope (a fully automatic Olympus microscope) at 10X [70].

### Photosynthetic pigments estimation

The chlorophyll and carotenoid content of 110-day-old fresh *Vicia faba* leaves was determined spectrophotometrically. Following standard protocols, chlorophyll a and b were quantified utilizing the technique outlined by [71], while carotenoids were measured following the protocol of Lichtenthaler HK, et al. [72]. Fresh leaf samples (100 mg) were homogenized in dimethyl sulfoxide (DMSO) and the resultant extract was filtered. The absorbance of the filtrate was recorded at 645, 663, and 470 nm using a UV/VIS spectrophotometer (Jenway, 7315). Pigment concentrations, expressed as µg/mL, were calculated using established equations adjusted for sample dilutions:

2 $$\begin{aligned}\:\mathbf{C}\mathbf{h}\mathbf{l}\mathbf{o}\mathbf{r}\mathbf{o}\mathbf{p}\mathbf{h}\mathbf{y}&\mathbf{l}\mathbf{l}\:\mathbf{a}\:(\boldsymbol{\upmu\:}\mathbf{g}\:/\:\mathbf{m}\mathbf{L})\:\\&=\mathbf{12.7}\:\mathbf{E}\mathbf{663}\:-\:\mathbf{2.69}\:\mathbf{E}\mathbf{645}\end{aligned}$$

3 $$\begin{aligned}\:\mathbf{C}\mathbf{h}\mathbf{l}\mathbf{o}\mathbf{r}\mathbf{o}\mathbf{p}\mathbf{h}\mathbf{y}\mathbf{l}\mathbf{l}\:\mathbf{b}\:&(\boldsymbol{\upmu\:}\mathbf{g}\:/\:\mathbf{m}\mathbf{L})\:\\&=\mathbf{22.9}\:\mathbf{E}\mathbf{645}\:-\:\mathbf{4.68}\:\mathbf{E}\mathbf{663}\:\end{aligned}$$

4 $$\begin{aligned}&\:\mathbf{C}\mathbf{a}\mathbf{r}\mathbf{o}\mathbf{t}\mathbf{e}\mathbf{n}\mathbf{o}\mathbf{i}\mathbf{d}\mathbf{s}\:(\boldsymbol{\upmu\:}\mathbf{g}\:/\:\mathbf{m}\mathbf{L})\:\\&=\frac{\left(\mathbf{1000}\:\mathbf{*}\:\:\mathbf{A}\mathbf{470}\right)-\left(\mathbf{1.82}\:\mathbf{*}\:\:\mathbf{c}\mathbf{h}\mathbf{l}\:\mathbf{a}\right)-\left(\mathbf{85.02}\:\mathbf{*}\:\:\mathbf{c}\mathbf{h}\mathbf{l}\:\mathbf{b}\right)\:\:}{\mathbf{198}}\end{aligned}$$

Where A: Absorbance of the pigment extract at 470 nm (where Carotenoids absorb strongly).

Chl a and Chl b: Concentrations of Chlorophyll a and b (in µg/mL) calculated from other equations.

1000, 1.82, 85.02, 198: constants derived from extinction coefficients.

(Fractions were calculated in milligrams per gram of fresh weight).

### Antioxidant assay

#### Non-enzymatic antioxidant assay and proline contents

To prepare *Vicia faba* leaf extract, 0.1 g of 110-day-old, dry, powdered leaves was shaken in 15 mL of methanol for one week at 30 °C. The resultant extraction mixture was filtered using Whatman number 1 filter paper. The collected filtrate (the crude extract) was then redissolved in a standardized volume of methanol, and stored at 4 °C for subsequent analysis [73].

##### Total phenolic contents determination

Using the Folin-Ciocalteu method, the total phenolic content of faba bean leaf extracts was assessed. The process involved combining 500 µL of the methanolic extract with a Folin-Ciocalteu reagent (10%) and Na_2_​CO_3_ (7.5%)​. A spectrophotometer was employed to measure the mixture’s absorbance at 765 nm following a 45-min incubation time at room temperature. The results are presented as milligrams of gallic acid equivalents per gram of dry leaf weight, based on a gallic acid standard curve. The experiment was performed three times to ensure accuracy [74].

##### Total flavonoids content determination

Following the colorimetric method described by Eberhardt MV, et al. [75]. The total flavonoid content was determined. A methanolic extract was mixed with distilled water. 5% Sodium nitrite (NaNO_2_) solution was added, followed by 10% aluminum chloride (AlCl_3_.6H_2_O) solution after 6 min. After 5 more min, sodium hydroxide (NaOH) was introduced, and flavonoid content was determined by measuring absorbance at 510 nm and was reported as milligrams of quercetin equivalents per gram of dry leaf weight.

##### Assessment of total proline in *Vicia Faba* leaves

Total proline content was determined as described by Bates LS, et al. [76]. 0.5 g of dry *Vicia faba* leaves was homogenized in 10 mL of distilled H_2_O and incubated at 90 °C for 60 min. The homogenate was filtered through Whatman 2 filter paper, the pellet was extracted twice, and the combined supernatant was raised to10 mL using distilled H_2_O. The reaction mixture containing 1000 µL of the extract, 1000 µL glacial acetic acid and 1000 µL Ninhydrin reagent (0.14 M), was incubated for 60 min at 100 °C, cooled in ice bath to stop the reaction and the absorbance was determined at 510 nm using a spectrophotometer. The standard curve was prepared by using different concentrations of L-proline. The L-proline content was expressed as milligrams of L-proline per gram of *Vicia faba* leaf dry weight (DW).

#### Enzymatic antioxidant assay

For preparation of enzymatic extract; 110-day-old fresh *Vicia faba* leaves were ground in 0.05 M phosphate buffer pH = 7. The resulting mixture was filtered, and the final volume of the enzymatic extract was adjusted to 5 mL [77].

##### Catalase assay (CAT)

CAT was estimated following the procedure described by [78]. The reaction mixture (430 µL) consisted of 20 µL of 0.2M H_2_O_2_, 400 µL potassium phosphate buffer (pH = 7) and 10 µL of enzyme extract. The reaction mixture was thoroughly mixed, and its absorbance at 240 nm was measured immediately, and at 10 to 20 s intervals. The time needed for the absorbance to decrease to 120 s was recorded.

##### Peroxidase assay (POD)

POD was determined according to Hammerschmidt R, et al. [79]. The reaction mixture consisted of 40 µL of 0.05 M pyrogallol, 400 µL 50 mM potassium phosphate buffer (pH = 7), 20 µL of H_2_O_2_ and 20 µL of enzyme extract measuring absorbance at 420 nm.

##### Polyphenol oxidase assay (PPO)

The analysis was performed using the method previously established by Assicot M, et al. [80]. PPO activity was determined by measuring absorbance at 420 nm. The reaction mixture contained 50 mM potassium phosphate buffer (pH = 7), 0.02 mM pyrogallol, and 100 µL of enzyme extract.

##### Phenylalanine Ammonia Lyase assay (PAL)

PAL was estimated following the procedure described by Khadilkar P, et al. [81]. The reaction mixture consisted of 3mM Phenylalanine (substrate), 100 µL of enzyme extract and 150 mM Tris HCL buffer (pH = 8.5) measuring absorbance at 270 nm.

5 $$\begin{aligned}&\:\mathrm{E}\mathrm{n}\mathrm{z}\mathrm{y}\mathrm{m}\mathrm{e}\:\mathrm{A}\mathrm{c}\mathrm{t}\mathrm{i}\mathrm{v}\mathrm{i}\mathrm{t}\mathrm{y}\:(\mathrm{U}\mathrm{n}\mathrm{i}\mathrm{t}/\mathrm{g}\:\mathrm{f}\mathrm{r}\mathrm{e}\mathrm{s}\mathrm{h}\:\mathrm{w}\mathrm{e}\mathrm{i}\mathrm{g}\mathrm{h}\mathrm{t})\:\\&=\frac{\mathrm{S}\mathrm{l}\mathrm{o}\mathrm{p}\mathrm{e}\times\:\mathrm{A}\mathrm{s}\mathrm{s}\mathrm{a}\mathrm{y}\:\mathrm{v}\mathrm{o}\mathrm{l}\mathrm{u}\mathrm{m}\mathrm{e}\:\left(\mathrm{m}\mathrm{L}\right)\times\:\mathrm{E}\mathrm{x}\mathrm{t}\mathrm{r}\mathrm{a}\mathrm{c}\mathrm{t}\:\mathrm{v}\mathrm{o}\mathrm{l}\mathrm{u}\mathrm{m}\mathrm{e}\:\left(\mathrm{m}\mathrm{L}\right)\:}{\mathrm{E}\mathrm{n}\mathrm{z}\mathrm{y}\mathrm{m}\mathrm{e}\:\mathrm{u}\mathrm{s}\mathrm{e}\:\left(\mathrm{m}\mathrm{L}\right)\times\:\mathrm{E}\:\times\:\mathrm{W}\mathrm{t}\:\mathrm{o}\mathrm{f}\:\mathrm{p}\mathrm{l}\mathrm{a}\mathrm{n}\mathrm{t}}\:\:\:\end{aligned}$$

*Slope*: Rate.

*Assay volume (mL)*: Cuvette volume.

*Extract volume (mL)*: 5 mL.

*Enzyme used (mL)*: Enzyme extract used volume.

*E*: Constant (for CAT: 34.9, POD: 12, PPO: 12 and PAL: 19.73).

*Wt of plant*: Weight of plant used for preparation of enzymatic extract (0.5 g).

### Biological features of soil (Soil fertility)

#### Total bacterial count & total halophytic bacterial count estimation

Colony Forming Unit (CFU) assay was used to determine the total bacterial count in soil samples. Soil from the experimental plots was analyzed both before planting and 145 days after planting to assess the bacterial population after treatment. A soil solution was prepared as described before, 10µL of this solution was spread onto two types of LB agar plates: one with a standard salt concentration 170 mM NaCl concentration (1%) and one with a high salt concentration 1700 mM NaCl concentration (10%) to specifically count halophytic bacteria, and the dishes were incubated at 28 °C for two days. The number of colonies were counted. Crowded colonies on plates were diluted to 10^− 2^ before counting to ensure an accurate count.

#### Dehydrogenase activity estimation

Dehydrogenase activity was estimated in soil samples collected both before planting and after 145 days of treatment. This was carried out in accordance with Mierzwa-Hersztek M, et al. [82] methodology. A soil sample was mixed with a 3% solution of 2,3,5-triphenyl tetrazolium chloride. Four days into the incubation at 27 °C, ethanol was introduced to the mixture, which was then vortexed and left to settle. The change of TTC to triphenyl-formazan (TPF), a measure of dehydrogenase activity, was quantified by measuring the absorbance of filtrate at 485 nm using a spectrophotometer. The findings were presented as microgram TPF per gram of dry soil per min [83].

### Yield parameters

For 145 day old at fruiting stage bean plants, three plants were taken out, and the following yield parameters were measured: straw yield, number and weight of pods per plant, length of pods, weight of seeds per plant, weight of 100 seeds and number of seeds per pod.

### Assessment of total proteins in *Vicia Faba* seeds

Sample of fresh *Vicia faba* dry seeds (0.2 g) was homogenized in 0.05 M (pH 8.0) Tris HCL buffer using precooled mortars and pestles. Homogenates were transferred to cold centrifuge tubes and centrifuged then the clear supernatants can be refrigerated at −20 °C for subsequent use or used right away for the protein test [84]. A blank containing an extraction buffer of 0.1 mL and 0.1 mL of each supernatant sample also were prepared. Then, for each tube, 3 mL of the Bradford reagent was added. The tubes were gently mixed to prevent foam production in the sample, which would reduce color yield. The absorbance at 595 nm was measured spectrophotometrically after 5 min and before 1 h. The net absorbances at 595 nm were subjected to regression analysis for each albumin standard (known concentration vs. absorbance). Using a standard curve derived from bovine serum albumin (BSA), the protein concentration was determined. For each unknown sample, the protein concentrations (expressed in samples mg/g in fresh weight) were calculated [85].

### Statistical analysis

Statistical analyses were performed using CoStat (version 6.450) and Microsoft Excel 2016. The experiment followed a randomized block design. One-way and two-way ANOVA were used to analyze the data, with mean averages compared using Tukey’s test at *p* ≤ 0.05. Finally, Pearson correlation coefficients were calculated and presented in a matrix to analyze relationships between variables.

## Results

### Impact of bacterial seed priming on *Vicia Faba* growth and survival

Increasing NaCl concentration to 150 mM (25% seawater) negatively affects seed germination by both decreasing germination rates and raising instances of pre- and post-emergence damping-off. Seeds inoculated with the bacteria *P.s* and the B mix achieved a maximum germination rate of 100% when cultivated in freshwater, wastewater and almost all NaCl concentration except *P.s* at a concentration of 30 mM NaCl showed 98.67%±0.02, but both *P.s* and B mix significantly improved germination percentages compared to the control group at freshwater, wastewater, 30, 90 and 150 mM NaCl concentration of the values 99%±0.01, 95%±0.05, 95%±0.05, 85%±0.05 and 60%±0.1, respectively (Table 1).

**Table 1:** Germination percentage, pre-emergency damping off and post-emergency damping off *Vicia Faba* L. Mariout-2 using single; *B.l*,* S.s*, and *P.s* and a mixture; B mix of halo-endophytic bacteria and irrigation with different concentrations of seawater after 14 days old

Irrigation	Treatments	%G	pre-emergence damping off	post-emergence damping off
Freshwater	Control	99%±0.01 ^ab^	1%±0.01 ^c^	16.67%±0.06 ^ghij^
	***B.l***	100%±0 ^a^	6.67%±0.06 ^bc^	0.67%±0.01 ^k^
	***S.s***	93.33%±0.06 ^abc^	6.67%±0.06 ^bc^	0.33%±0.01 ^k^
	***P.s***	100%±0 ^a^	0%±0 ^c^	5%±0.05 ^jk^
	B mix	100%±0 ^a^	0%±0 ^c^	5.33%±0.05 ^jk^
Wastewater	Control	95%±0.05 ^abc^	5%±0.05 ^bc^	5%±0.05 ^jk^
	***B.l***	96.67%±0.06 ^ab^	3.33%±0.06 ^bc^	0%±0 ^k^
	***S.s***	100%±0 ^a^	0%±0 ^c^	0%±0 ^k^
	***P.s***	100%±0 ^a^	0%±0 ^c^	30%±0.1 ^defg^
	B mix	100%±0 ^a^	0%±0 ^c^	10%±0.1 ^hijk^
30 mM NaCl conc	Control	95%±0.05 ^abc^	5%±0.05 ^bc^	25%±0.05 ^efgh^
	***B.l***	99.67%±0.01 ^a^	1%±0.01 ^c^	4.67%±0.05 ^jk^
	***S.s***	100%±0 ^a^	0%±0 ^c^	5%±0.05 ^jk^
	***P.s***	98.67%±0.02 ^ab^	1.33%±0.02 ^c^	20%±0.1 ^fghij^
	B mix	100%±0 ^a^	0%±0 ^c^	0%±0 ^k^
90 mM NaCl conc	Control	85%±0.05 ^bc^	15%±0.05 ^bc^	85%±0.05 ^ab^
	***B.l***	90%±0.1 ^abc^	10%±0.1 ^bc^	15%±0.05 ^ghijk^
	***S.s***	96.67%±0.06 ^ab^	3.33%±0.06 ^bc^	8.33%±0.03 ^ijk^
	***P.s***	100%±0 ^a^	0%±0 ^c^	0%±0 ^k^
	B mix	100%±0 ^a^	0%±0 ^c^	0%±0 ^k^
150 mM NaCl conc	Control	60%±0.1 ^d^	40%±0.1 ^a^	100%±0 ^a^
	***B.l***	100%±0 ^a^	0%±0 ^c^	0.33%±0.01 ^k^
	***S.s***	99.33%±0.01 ^ab^	0.67%±0.01 ^c^	0%±0 ^k^
	***P.s***	100%±0 ^a^	0%±0 ^c^	0%±0 ^k^
	B mix	100%±0 ^a^	0%±0 ^c^	0%±0 ^k^
Effect of inoculation with halo-endophytic bacteria				
Control		86.8 ^c^	13.2 ^ab^	46.33 ^a^
B.l		97.26 ^ab^	8.2 ^bc^	4.13 ^cd^
S.s		97.86 ^ab^	4.2 ^cd^	2.73 ^d^
P.s		99.73 ^a^	0.26 ^d^	11 ^b^
B mix		100 ^a^	0 ^d^	3.06 ^d^
Effect of irrigation with different concentration of seawater				
Freshwater		98.38 ^a^	2.57 ^c^	12.09 ^d^
Wastewater		94.76 ^ab^	5.23 ^bc^	31.66 ^ab^
30 mM NaCl conc		98.38 ^a^	1.71 ^c^	21.61 ^c^
90 mM NaCl conc		86.42 ^c^	14.04 ^a^	34.76 ^a^
150 mM NaCl conc		91.33 ^b^	9.14 ^b^	29.33 ^b^

*Bl:* B. licheniformis DW4, *Ss:* *S. sesuvii *DW5, *Ps:* *P. suwonensis* DW7 and B mix: *B. licheniformis* DW4 + S. sesuvii DW5 + P. suwonensis DW7. Value: mean ± S.D, Means shared the same letter(s) are not significantly different, Tuckey test (*p* ≤ 0.05). The letters represent the ranking of groups based on significant differences, where 'a' denotes the highest mean value and subsequent letters denote significantly lower values in descending order.

Generally, seeds inoculated with bacteria *P.s* and B mix at freshwater, wastewater and almost all concentrations of NaCl showed no pre-emergency damping off except *P.s* at 30 mM NaCl concentration showed 1.33%±0.02 compared to *B.l* and *S.s* of 6.67%±0.06 at freshwater, control of 5%±0.05 at 30 mM NaCl concentration and control of 40%±0.1 at 150 mM NaCl concentration which showed the highest pre-emergency damping off.

Seeds inoculated with B mix at all concentrations of NaCl showed no post-emergency damping off at irrigation with, wastewater and all concentrations of NaCl compared to control at 90 and 150 mM NaCl concentration of 85%±0.05 and 100%±0, respectively. Under wastewater irrigation, untreated seeds (control) did not survive (Table 1).

### Faba seed biopriming’s impact on parameters of growth under salt stress

Inoculation of faba bean plants with single or mixed cultures of bacteria was tested to determine the impact on plant growth performance under saline soil conditions. The plants were irrigated with different concentrations of seawater, and the effects were measured by assessing changes in faba branch number, leaf number, root and shoot length, the fresh and dry weight of both roots and shoots and plant height for each treatment.

**Fig. 3:**
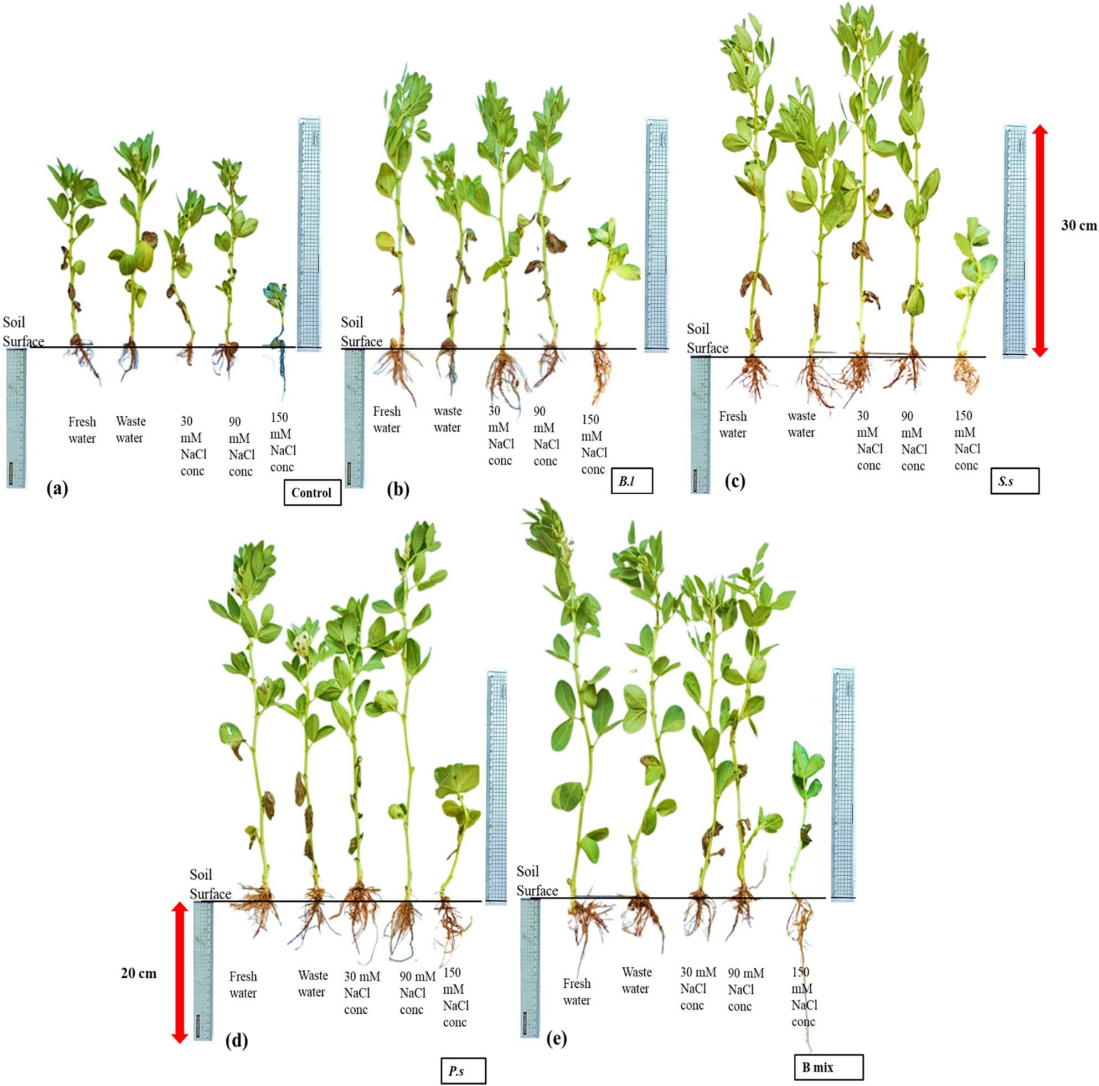
*Vicia faba* plants inoculated with different halo-endophytic bacteria; *B.l, S.s, P.s* and B mix compared to control, grown in salt-affected soil and irrigated with different seawater concentrations after 45 days.a: control,b (*Bl):* *B.licheniformis* DW4, c (*Ss*): *S. sesuvii* DW5, d (*Ps*): *P. suwonensis* DW7ande (B mix): *B.licheniformis* DW4 *+ S. sesuvii* DW5 *+* *P. suwonensis* DW7

**Fig. 4:**
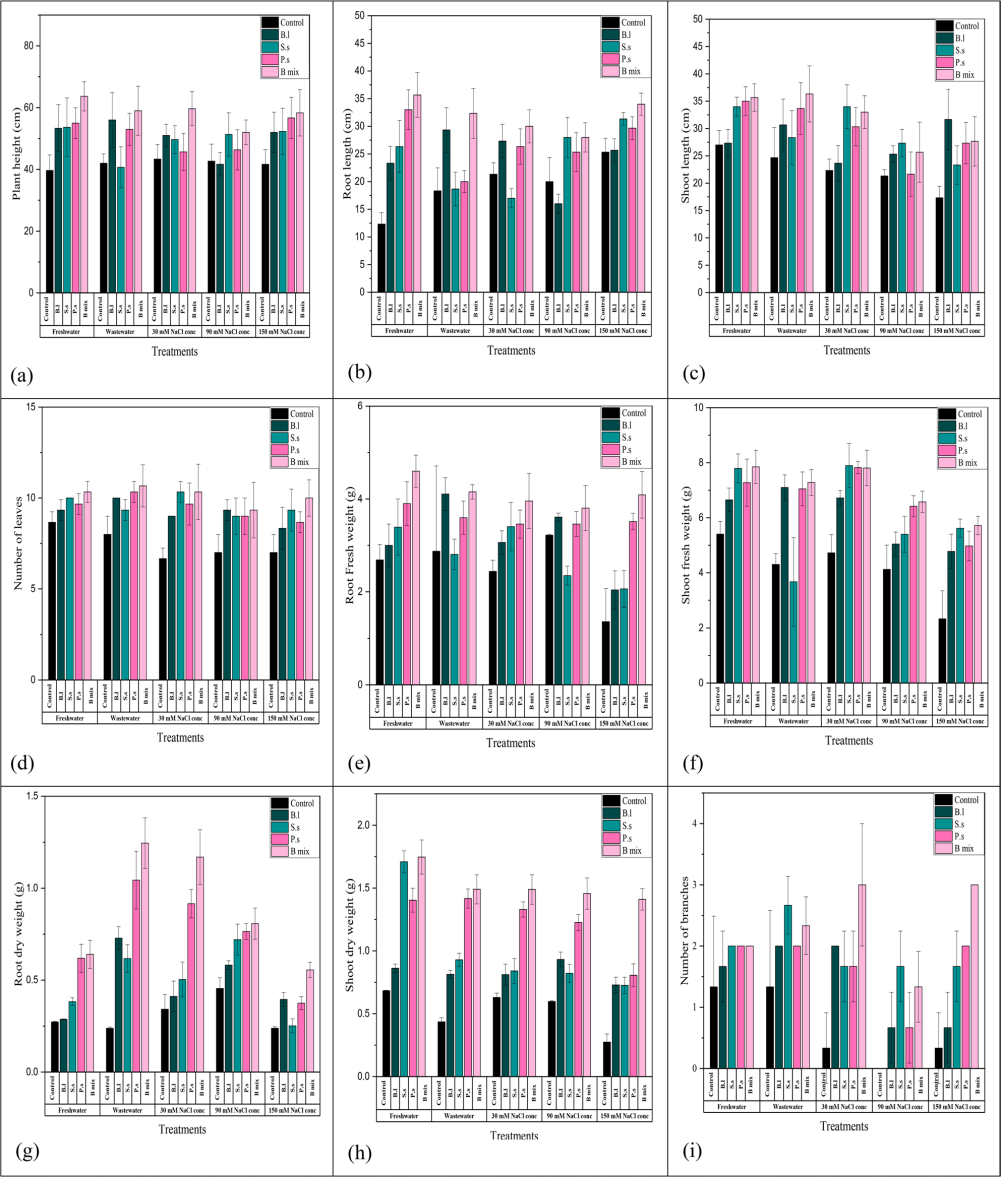
Histogram of growth parameters of *Vicia faba* with and without halo-endophytic bacteria after 45 days. a: plant height, b: root length, c: shoot length, d: number of leaves, e: root fresh weight, f: shoot fresh weight, g: root dry weight, h: shoot dry weight and i: number of branches. C: control,* Bl: B. licheniformis* DW4, *Ss: S. sesuvii* DW5, *Ps*:* P. suwonensis* DW7andB mix: *B.licheniformis *DW4* + S. sesuvii *DW5* + P. suwonensis* DW7

Overall, bacterial treatments; *B.l*, *S.s*,* P.s* and B mix displayed increased rates for all parameters of growth in comparison to the untreated control after 45 (vegetative stage) and 110 (flowering stage) cultivation days and B mix was found to be the most effective among all treatments, generally followed by *P. suwonensis* DW7, *S. sesuvii* DW5, and *B. licheniformis* DW4. After 45 days old (Figs. 3 and 4; Table 2), the maximum plant height was obtained in seeds inoculated with treatment B mix at freshwater, wastewater, 30, 90 and 150 mM NaCl concentration and the highest was 63.33 ± 5.03 cm at freshwater. The maximum root length and shoot length were obtained in treated seeds with B mix at freshwater, wastewater, 30, 90 and 150 mM NaCl concentration and the highest root length were 34 ± 2 cm at 150 mM NaCl concentration and the shoot length was 55.33 ± 7.02 cm at freshwater. The highest number of leaves was obtained in treated seeds with B mix at freshwater, wastewater, 30, 90 and 150 mM NaCl concentration and the highest number of leaves was 16 ± 1 at freshwater. The maximum root shoot fresh weight was obtained in treated seeds with B mix at freshwater, wastewater, 30, 90 and 150 mM NaCl concentration and the fresh weight of the roots and shoots that were the highest were 4.09 ± 0.5 g at 150 mM NaCl concentration and 15.13 ± 1.21 g at freshwater. The maximum root and shoot DWs were obtained in treated seeds with B mix at freshwater, wastewater, 30, 90 and 150 mM NaCl concentration and the highest root and shoot DWs were 0.56 ± 0.042 g at 150 mM NaCl concentration and 1.6 ± 0.1 g at wastewater. The maximum number of branches was obtained in seeds inoculated with B mix at freshwater, wastewater 30, 90 and 150 mM NaCl concentration and 3 ± 0 at 150 mM NaCl concentration was the greatest number of branches.

**Table 2:**
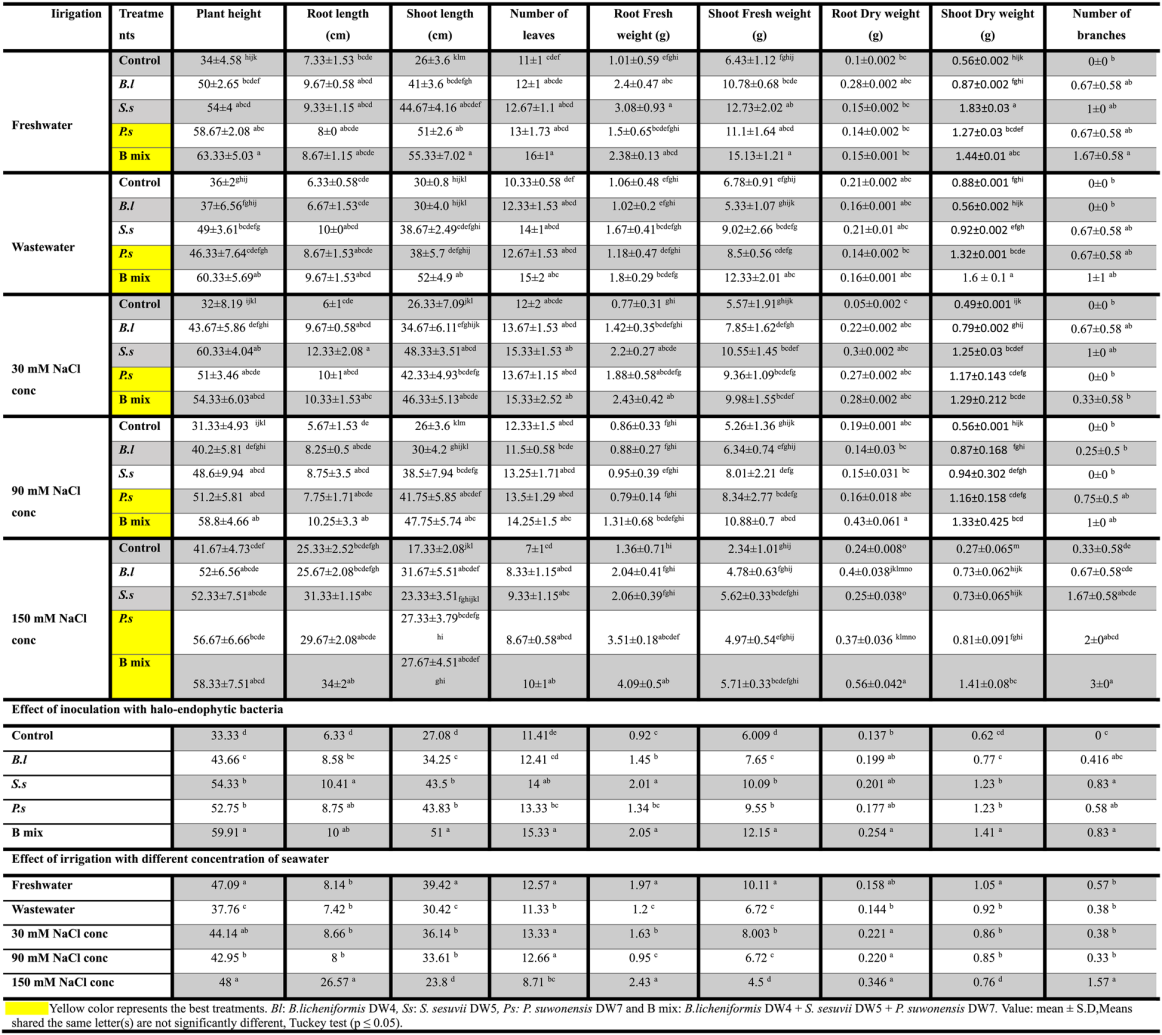
Different growth parameters of *Vicia Faba* treated with single; *B.l*,* S.s*, and *P.s* and a mixture; B mix of halo-endophytic bacteria and cultivated in salt-affected soil after 45 days

**Fig. 5:**
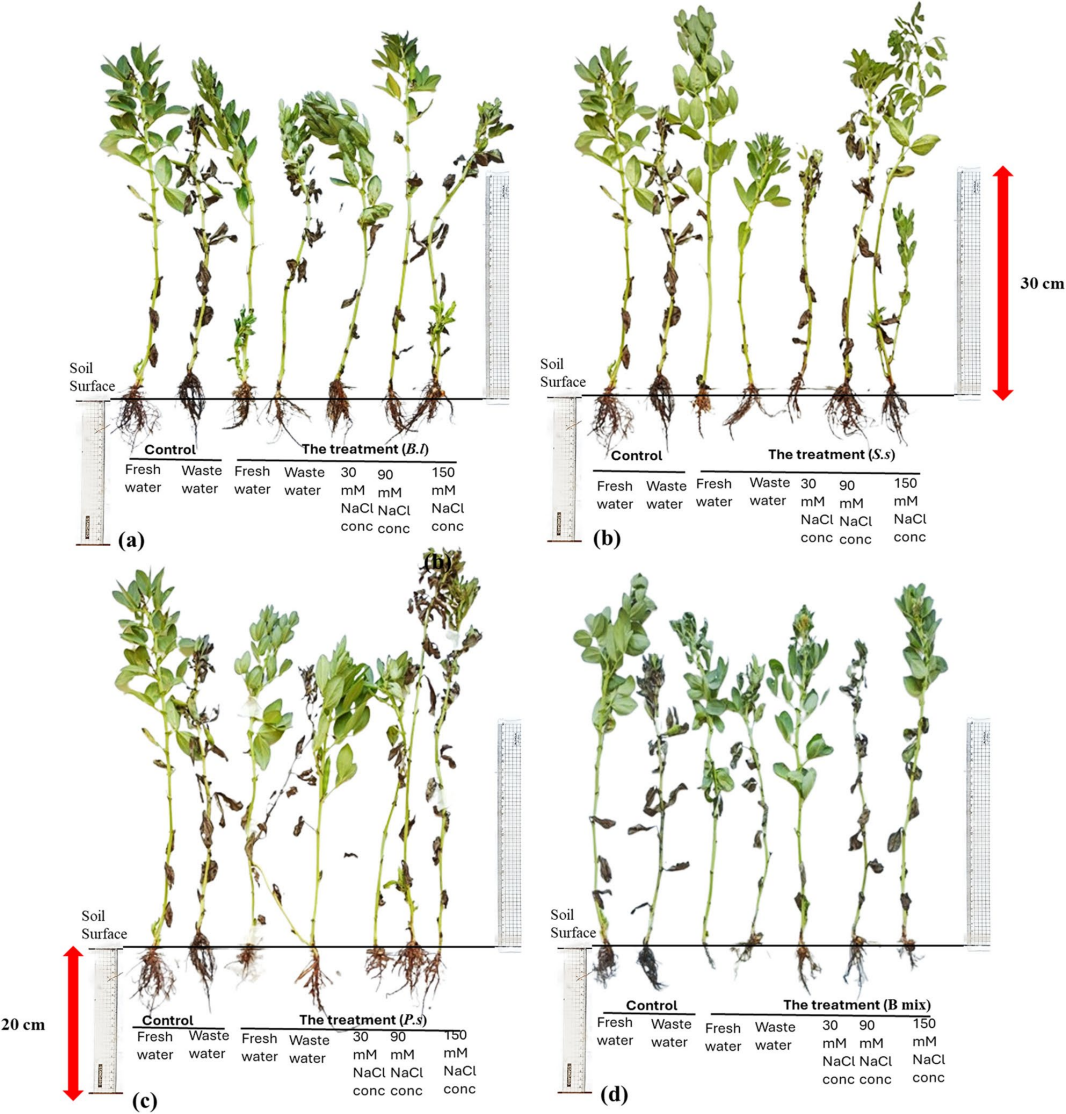
*Vicia faba *plants inoculated with different halo-endophytic bacteria; *B.l, S.s, P.s *and B mix compared to control, grown in salt-affected soil and irrigated with different seawater concentrations after 110 days.a (*Bl*)*: B. licheniformis* DW4, b (*Ss*)*: S. sesuvii* DW5,c (*Ps*):* P. suwonensis* DW7and d(B mix): *B.licheniformis *DW4* + S. sesuvii *DW5* + P. suwonensis* DW7

**Fig. 6:**
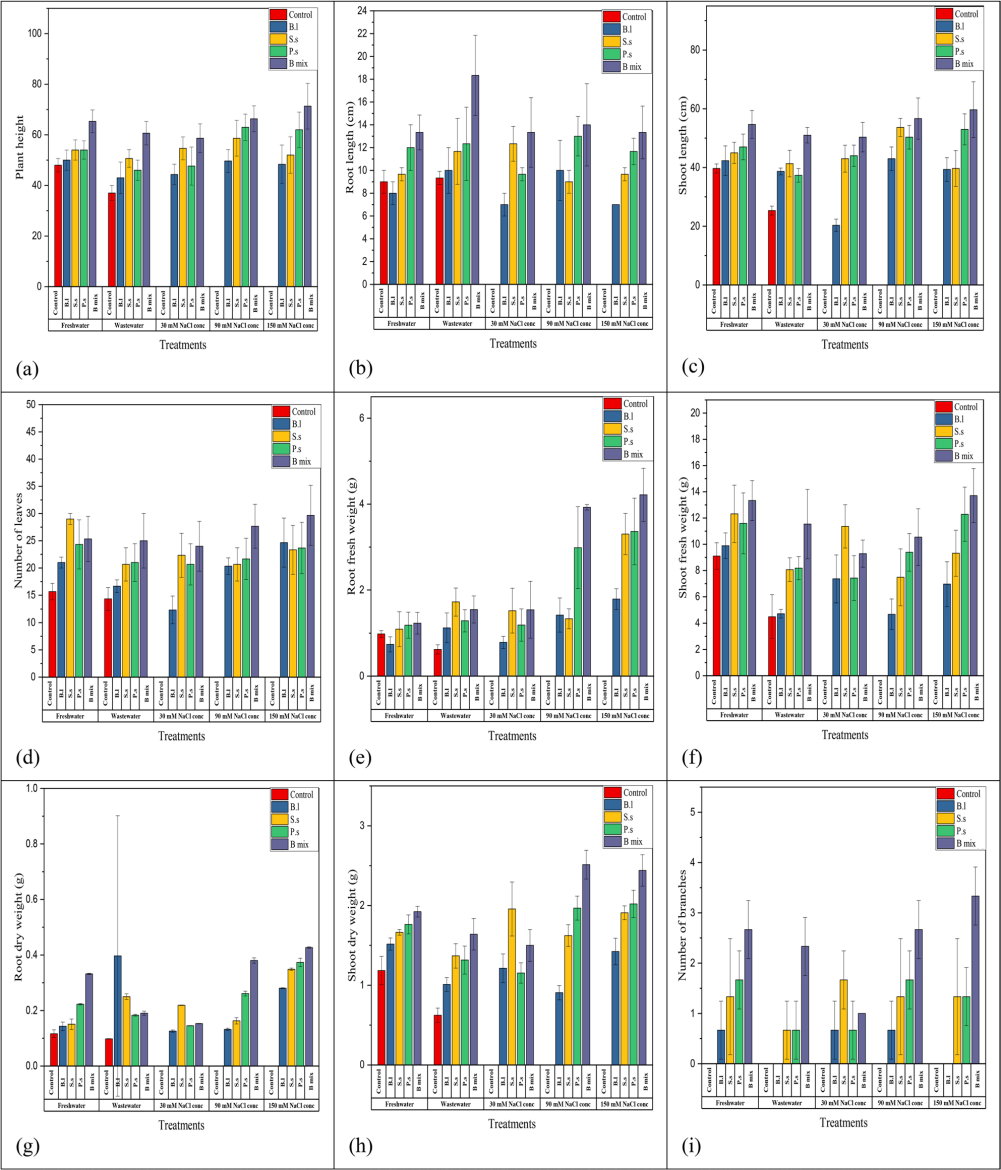
Histogram of growth parameters of *Vicia faba* with and without halo-endophytic bacteria after 110 days. a: plant height, b: root length, c: shoot length, d: number of leaves, e: root fresh weight, f: shoot fresh weight, g: root dry weight, h: shoot dry weight and i: number of branches. C: control,* Bl: B. licheniformis* DW4, *Ss: S. sesuvii* DW5, *Ps*:* P. suwonensis* DW7andB mix: *B.licheniformis *DW4* + S. sesuvii *DW5* + P. suwonensis* DW7

After 110 days (Figs. 5 and 6; Table 3), the highest four treatments; *B.l*, *S.s*,* P.s* and B mix beside the freshwater and wastewater controls (controls at 30, 90 and 150 mM NaCl concentration were died at 110 days old) were chosen for the determination of parameters of growth after 110 days of planting, B mix-treated seeds produced the maximum plant height at freshwater, wastewater, 30, 90 and 150 mM NaCl concentration and the highest were 71.33 ± 9.02 cm at 150 mM NaCl concentration. Seeds inoculated with B mix at freshwater, wastewater, 30, 90 and 150 mM NaCl concentration showed the highest root length and shoot length, and the highest root length was 18.33 ± 3.51 cm at wastewater, and shoot length was 59.67 ± 9.5 cm at 150 mM NaCl concentration. The maximum number of leaves was obtained in treated seeds with B mix at freshwater, wastewater, 30, 90 and 150 mM NaCl concentration and the highest number of leaves was 29.67 ± 5.51 at 150 mM NaCl concentration. The maximum root shoot fresh weight were obtained in treated seeds with B mix at freshwater, wastewater, 30, 90 and 150 mM NaCl concentration and the highest root and shoot fresh weights were 4.22 ± 0.62 g and 13.71 ± 2.06 g at 150 mM NaCl concentration.

**Table 3:**
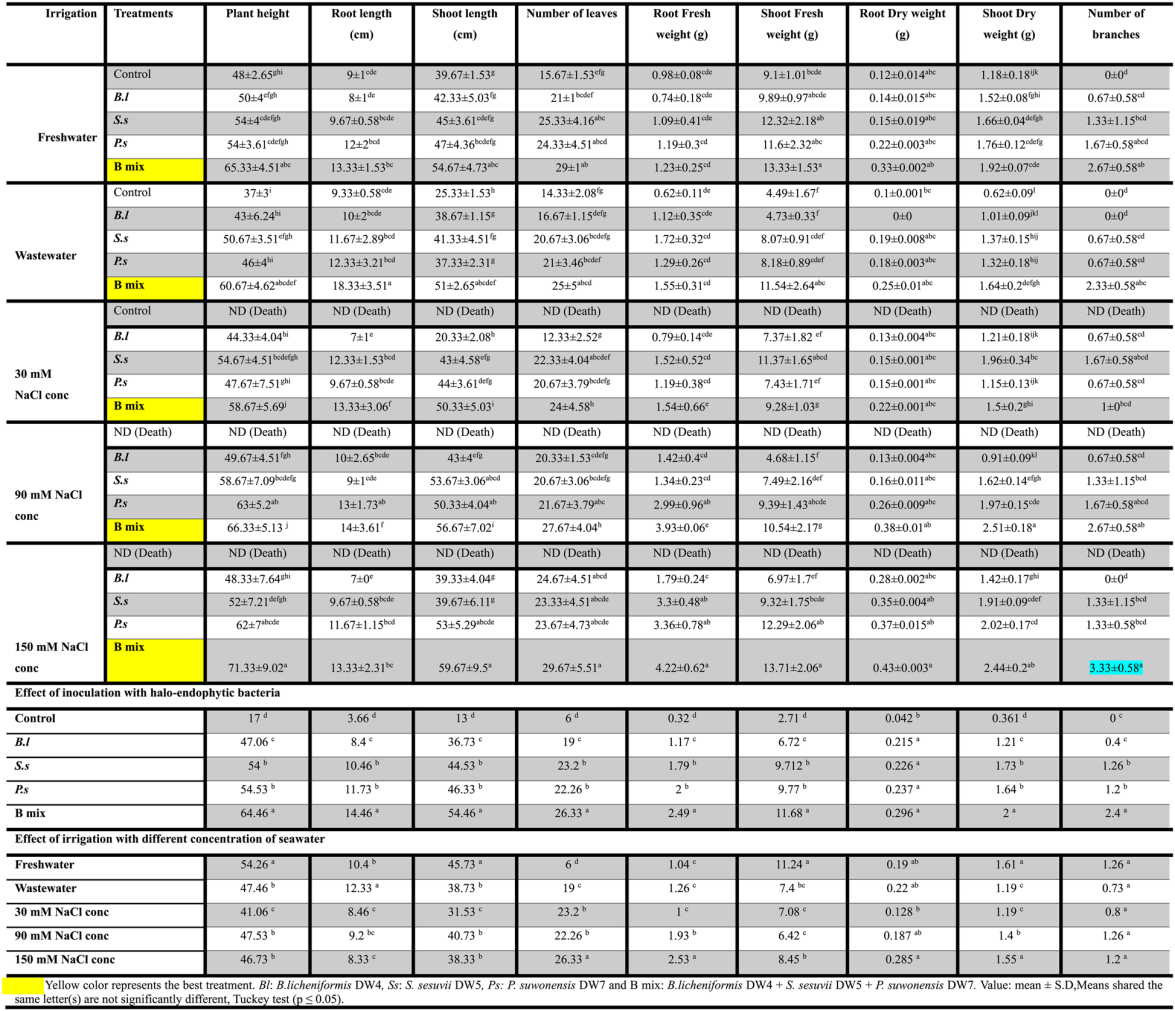
Different growth parameters of *Vicia Faba* treated with single; *B.l*,* S.s and P.s* and a mixture; B mix of halo-endophytic bacteria and cultivated in salt-affected soil after 110 days

The maximum DW of root and shoot were obtained in treated seeds with B mix at freshwater, wastewater, 30, 90 and 150 mM NaCl concentration and the highest root DW was 0.43 ± 0.003 g at 150 mM NaCl concentration and the shoot DW was 2.51 ± 0.18 g at 90 mM NaCl concentration. The maximum number of branches was obtained in treated seeds with B mix at freshwater, wastewater, 30, 90 and 150 mM NaCl concentration and the highest number of branches was 3.33 ± 0.58 at 150 mM NaCl.

### The impact of biopriming Faba beans on root nodulation

Visual analysis of the roots revealed that there was no nodulation on the non-treated plants (control). On the other hand, bacterial treatments; *B.l*, *S.s*,* P.s* and B mix showed nodulation after 40, 60, 88 and 110 days and B mix was found to be the most effective among all treatments, generally followed by *P.s*, *S.s*, and *B.l*.

**Fig. 7:**
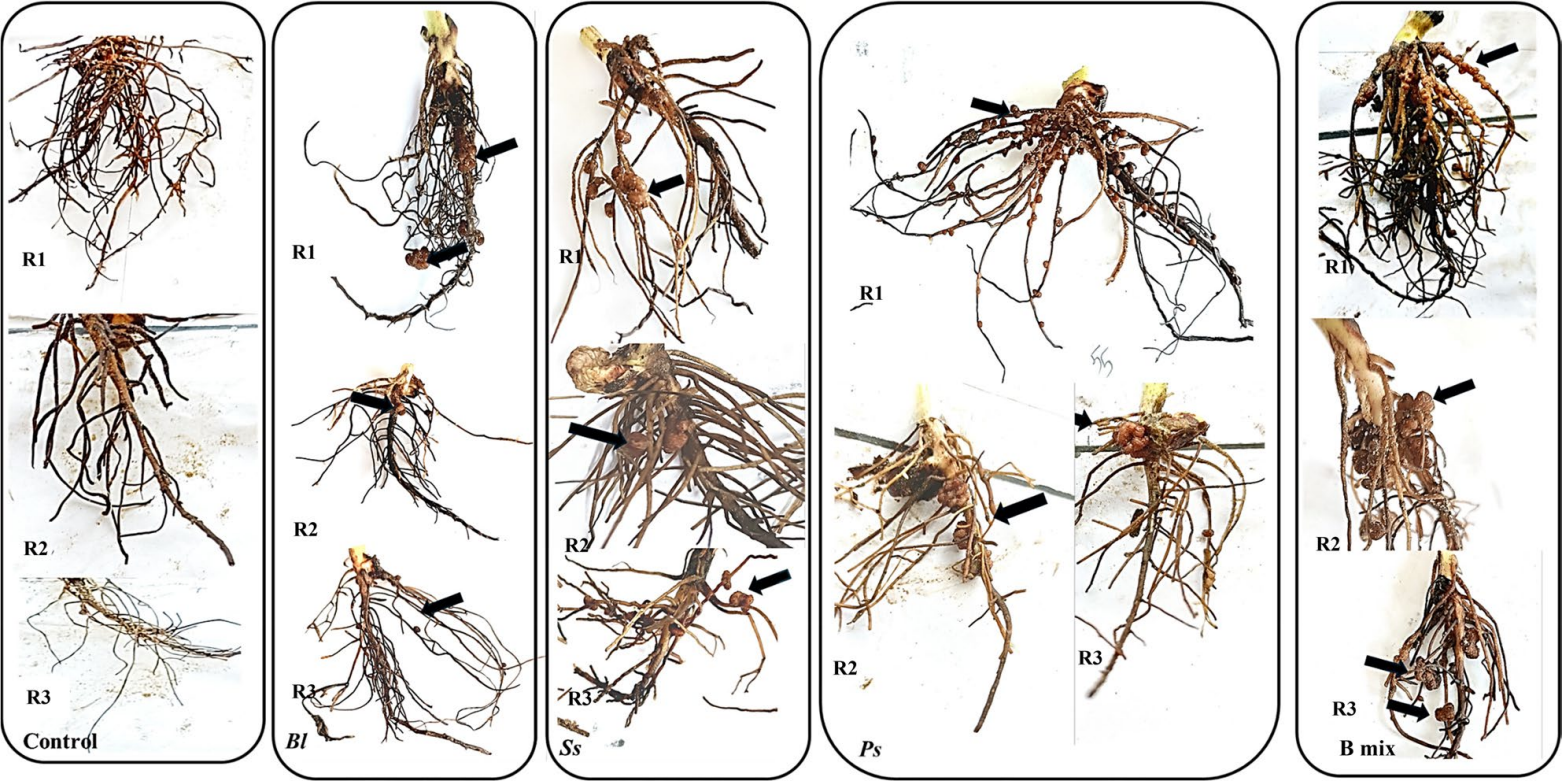
Root nodules of *Vicia faba *inoculated with different halo-endophytic bacteria; *B.l, S.s, P.s *and B mix compared to control, grown in salt-affected soil and irrigated with different seawater concentrations after 110 days.* Bl: B.licheniformis* DW4, *Ss: S. sesuvii* DW5,* Ps*:* P. suwonensis* DW7andB mix: *B.licheniformis *DW4* + S. sesuvii *DW5* + P. suwonensis* DW7. R1, R2 and R3: Replicates

After 45 days (Fig. 7; Table 4), seeds inoculated with bacteria B mix at freshwater, wastewater and 30, 90 and 150 mM NaCl concentration showed the maximum number and weight of nodules and the highest was 41 ± 1 at 30 mM NaCl concentration and 0.158 ± 0.0137 g at freshwater, respectively. After 110 days (Fig. 7; Table 4), the maximum number and weight of nodules were obtained in seeds treatment B mix at freshwater, wastewater and 30, 90 and 150 mM NaCl concentration and the highest number of nodules and weight of nodules were 50 ± 3 and 0.67 ± 0.03 g at 150 mM NaCl concentration.

**Table 4:** The number and weight of root nodules of *Vicia Faba* treated with singles; *B.l*,* S*.*s* and *P.s* and a mixture; B mix of halo-endophytic bacteria and cultivated in salt-affected soil after 45 and 110 days

Iirigation	Treatments	45 days			110 days	
		Number of nodules		Weight of nodules (g)	Number of nodules	Weight of nodules (g)
Freshwater	Control	ND		ND	ND	ND
	***B.l***	6.67 ± 0.58^efg^		0.121 ± 0.009^c^	5.67 ± 4.04^ghi^	0.17 ± 0.02^efg^
	***S.s***	14 ± 2.65^c^		0.151 ± 0.0085^ab^	9 ± 1^efg^	0.15 ± 0^fgh^
	***P.s***	7 ± 1^ef^		0.129 ± 0.022^bc^	4.67 ± 3.51^ghi^	0.04 ± 0.01^j^
	B mix	34.67 ± 1.53^b^		0.158 ± 0.0137^a^	45 ± 1^bc^	0.2 ± 0.1^def^
Wastewater	Control	ND		ND	ND	ND
	***B.l***	1.67 ± 0.58^hijk^		0.042 ± 0.0076^ghij^	3.33 ± 3.51^ghi^	0.14 ± 0.01^fghi^
	***S.s***	1.33 ± 0.58^ijk^		0.061 ± 0.0085^efg^	4 ± 2.65^ghi^	0.25 ± 0.03^de^
	***P.s***	1.67 ± 0.58^hijk^		0.063 ± 0.0064^efg^	1.33 ± 1.53^hi^	0.04 ± 0.02^j^
	B mix	4.67 ± 0.58^efghi^		0.137 ± 0.0246^abc^	11 ± 1^ef^	0.08 ± 0.02^ghij^
30 mM NaCl conc	Control	ND		ND	ND	ND
	***B.l***	3 ± 0^ghijk^		0.004 ± 0.001^mn^	1 ± 1^hi^	0.01 ± 0^j^
	***S.s***	7.67 ± 2.52^de^		0.05 ± 0.001^fghi^	69 ± 1^a^	0.48 ± 0.03^b^
	***P.s***	1.33 ± 0.58^ijk^		0.038 ± 0.0137^ghijk^	4 ± 1^ghi^	0.06 ± 0.03^ij^
	B mix	41 ± 1^a^		0.076 ± 0.0121^de^	4.33 ± 0.58^ghi^	0.2 ± 0.02^def^
90 mM NaCl conc	Control	ND		ND	ND	ND
	***B.l***	1.67 ± 0.58^hijk^		0.002 ± 0.001^n^	2.67 ± 0.58^hi^	0.03 ± 0.02^j^
	***S.s***	0.67 ± 0.58^jk^	0.002 ± 0.001^n^		35 ± 2^d^	0.26 ± 0.04^d^
	***P.s***	4 ± 0^efghij^	0.061 ± 0.009^efg^		39.67 ± 4.51^cd^	0.36 ± 0.04^c^
	B mix	4.67 ± 2.89^efghi^	0.07 ± 0.0095^def^		14.67 ± 1.53^e^	0.39 ± 0.03^c^
150 mM NaCl conc	Control	ND	ND		ND	ND
	***B.l***	0 ± 0^k^	0 ± 0^n^		0 ± 0^i^	0 ± 0^j^
	***S.s***	1.67 ± 0.58^hijk^	0.04 ± 0.01^ghij^		2.33 ± 0.58^hi^	0.08 ± 0^hij^
	***P.s***	3.33 ± 3.06^fghijk^	0.03 ± 0.01^hijkl^		6.33 ± 1.15^fgh^	0.43 ± 0.04^bc^
	B mix	11 ± 1^cd^	0.053 ± 0.0153^efgh^		50 ± 3^b^	0.67 ± 0.03^a^
Control		0.00 ^e^	0.00 ^d^		0.00 ^d^	0.00 ^e^
B.l		2.26 ^cd^	0.03 ^c^		1.8 ^d^	0.061 ^d^
S.s		3.46 ^c^	0.06 ^b^		15.46 ^c^	0.185 ^c^
P.s		8.4 ^b^	0.063 ^b^		19.93 ^b^	0.253 ^b^
B mix		15.86 ^a^	0.093 ^a^		24.66 ^a^	0.298 ^a^
Freshwater		10.66 ^a^	0.11 ^a^		13.4 ^c^	0.11 ^c^
Wastewater		1.71 ^c^	0.057 ^b^		3.93 ^d^	0.102 ^c^
30 mM NaCl conc		8.8 ^b^	0.032 ^c^		15.66 ^b^	0.149 ^b^
90 mM NaCl conc		1.57 ^c^	0.019 ^d^		18.4 ^a^	0.209 ^a^
150 mM NaCl conc		2.57 ^c^	0.018 ^d^		11.73 ^c^	0.235 ^a^

### Effect of faba seeds biopriming on root anatomy

**Fig. 8:**
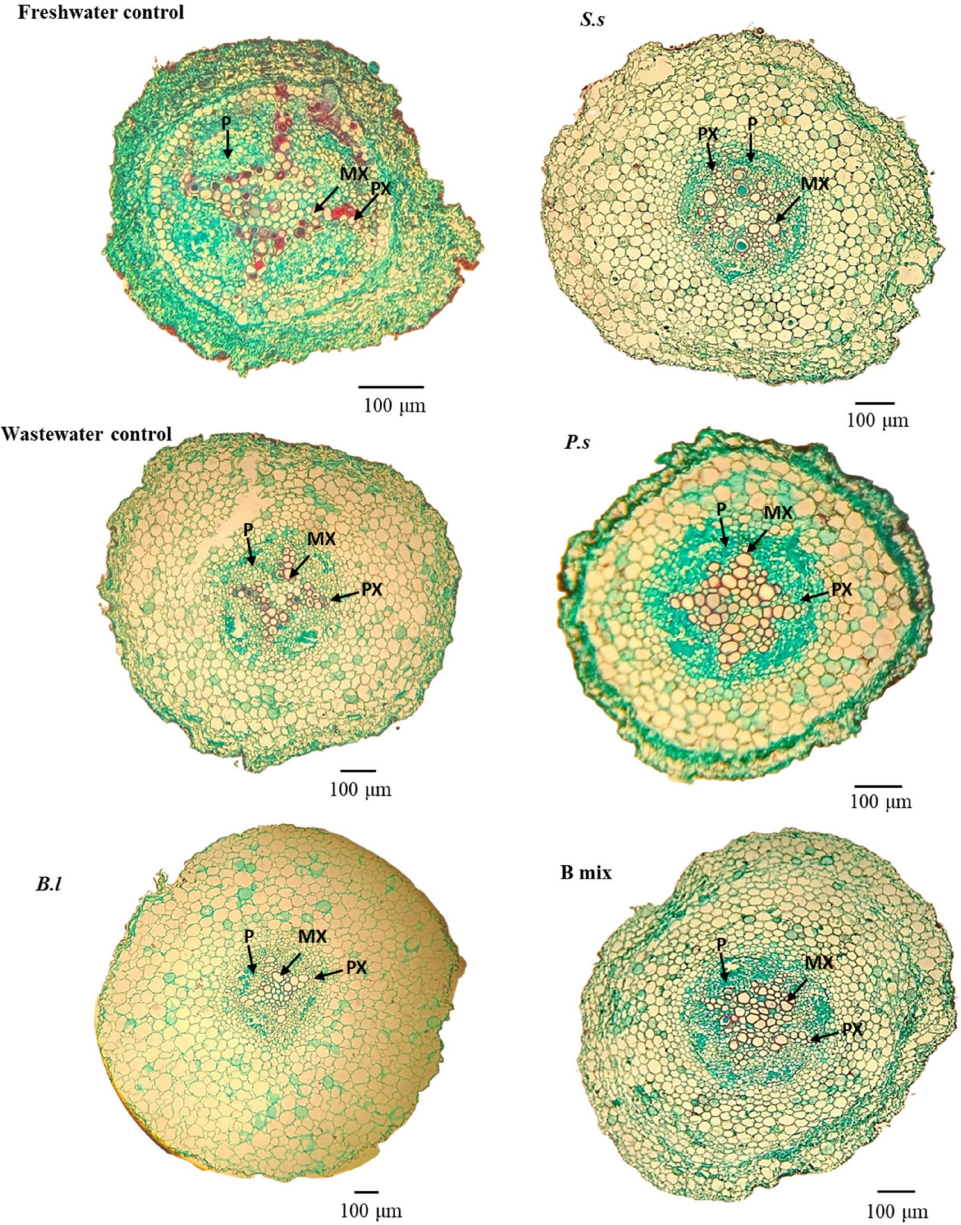
Transvers sections of *Vicia faba* L. Mariout-2 roots treated with different halo-endophytic bacteria; *B.l, S.s, P.s *and B mix compared to control after 110 days of growth.* B.l*: *B. licheniformis *DW4, *Ss: S. sesuvii* DW5, *Ps*:* P. suwonensis* DW7andB mix: *B. licheniformis *DW4* + S. sesuvii *DW5 *+ P. suwonensis* DW7. P: phloem, PX: Proto-xylem and MX: Meta-xylem

Roots from *Vicia faba* plants exposed to bacterial treatments; *B.l*, *S.s*,* P.s* and B mix at the highest NaCl concentration (150 mM) applied in watering system beside freshwater and wastewater controls were used in light microscopy of transverse sections (T.S) after 110 days of planting. All treatments were affected by salt stress and this was obviously in the change of the anatomical structure of roots compared to control (Fig. 8).

The size of the vascular cylinder was affected greatly by the type of bacterial treatments, where freshwater control ˃ *P.s* ˃ *S.s* ˃ B mix ˃ wastewater control ˃ *B.l*. The amount of xylem and phloem was affected greatly by the type of bacterial treatments, where *P.s* and B mix were more or less the same to freshwater control, whereas *S.s* and *B.l* were looked like wastewater control. In *P.s*, a complete ring of one or two layers of thick-walled cells under the piliferous layer was formed (Fig. 8).

### Effect of faba seeds biopriming on photosynthetic pigments content under salt stress

The four treatments; *B.l*, *S.s*,* P.s* and B mix from previous experiments after 110 days of planting were chosen at 150 mM for evaluation of the changes in photosynthetic pigments content of *Vicia faba* beside fresh and wastewater controls as shown in Table 5. All treatments under salt stress of 150 mM NaCl concentration showed total chlorophyll, chlorophyll a, chlorophyll b, total pigments and carotenoids contents higher than freshwater control (positive control), and wastewater control (Table 5).

**Table 5:** Effect of Faba seed biopriming with different halo-endophytic bacteria; *B.l*,* S.s*,* P.s* and B mix of 150 mM NaCl concentration compared to control and salt stress on photosynthetic pigments content; chlorophyll a, chlorophyll b, total chlorophyll, carotenoids and total pigments

Treatments	Chl a (mg/g f.w.)	Chl b (mg/g f.w.)	Total chlorophyll (mg/g f.w)	Carotenoids (mg/g f.w)	Total pigments (mg/g f.w)
Freshwater control	0.334 ± 0.267 ^b^	0.401 ± 0.407^abc^	0.734 ± 0.523 ^b^	0.094 ± 0.063 ^ab^	1.47 ± 1.05 ^b^
Wastewater control	0.292 ± 0.429 ^b^	0.213 ± 0.248 ^bc^	0.505 ± 0.588 ^b^	0.071 ± 0.06 ^b^	1.01 ± 1.18 ^b^
B.l	1.46 ± 0.12 ^a^	0.664 ± 0.172 ^abc^	2.124 ± 0.244 ^a^	0.357 ± 0.088 ^ab^	4.25 ± 0.49 ^a^
S.s	1.792 ± 0.347 ^a^	1.004 ± 0.234 ^a^	2.796 ± 0.452 ^a^	0.243 ± 0.133 ^ab^	5.59 ± 0.9 ^a^
P.s	1.96 ± 0.389 ^a^	0.61 ± 0.238 ^abc^	2.57 ± 0.388 ^a^	0.382 ± 0.137 ^a^	5.14 ± 0.78 ^a^
B mix	2.044 ± 0.429 ^a^	0.851 ± 0.603^ab^	2.895 ± 0.39 ^a^	0.267 ± 0.187 ^ab^	5.79 ± 0.78 ^a^

*Bl:* *B.licheniformis* DW4, *Ss:* *S. sesuvii* DW5, *Ps:* *P. suwonensis* DW7 and B mix: *B.licheniformis* DW4 + *S. sesuvii* DW5 + *P.* suwonensis DW7. Value: mean ± S.D, Means shared the same letter(s) are not significantly different, Tuckey test (*p* ≤ 0.05)

The maximum chlorophyll a, chlorophyll b and total chlorophyll contents were recorded in treatment B mix, *S.s* and B mix of 2.044 ± 0.429 mg/g fresh weight, 1.004 ± 0.234 and 2.895 ± 0.39 mg/g fresh weight, respectively, compared with the freshwater control showed a negative result. The maximum content of carotenoids and total pigments were recorded in treatment *P.s* and B mix of 0.382 ± 0.137 and 5.79 ± 0.78 mg/g fresh weight, compared with control freshwater, and wastewater controls, showed negative results (Table 5).

### Non-enzymatic antioxidants assay and proline contents of *Vicia faba *under salt stress after 110 days of planting

#### Non-enzymatic antioxidants

Total phenolics was determined using standard curve of gallic acid (GA), the results (Table 6) showed significant differences in all treatments as compared to freshwater and wastewater controls, the maximum content was 4.6 ± 0.09 and 3.98 ± 0.96 mg (GAE)/g DW, found in B mix and *P.s*. Total flavonoids (TF) were determined using the standard curve of quercetin (QE) and results were significant as expressed in Table 6. In comparison with freshwater and wastewater controls, the maximum content observed in B mix and *P.s* of 16.73 ± 2.17 and 14.2 ± 2.77 mg (QE)/g DW, respectively.

**Table 6:** Effect of different treatments with halo-endophytic bacteria; *B.l*,* S.s*,* P.s* and B mix and salt stress of 150 mM NaCl concentration compared to control on total phenolics, total flavonoids and proline contents in *Vicia Faba* and expressed as Unit/g fresh weight

Treatments	Total phenolic content (mg/g)	Total flavonoids content (mg/g)	Proline (mg/g)
Freshwater control	3.39 ± 0.91^ab^	11.03 ± 3.93^a^	1.83 ± 1.67^c^
Wastewater control	3.92 ± 1.06^ab^	13.82 ± 7.69^a^	2.59 ± 0.12^bc^
B.l	3.22 ± 0.65^ab^	9.16 ± 2.35^a^	4 ± 0.55^a^
S.s	3.29 ± 0.4^ab^	9.4 ± 1.15^a^	3.45 ± 0.68^abc^
P.s	3.98 ± 0.96^ab^	14.2 ± 2.77^a^	3.71 ± 0.7^abc^
B mix	4.6 ± 0.09^a^	16.73 ± 2.17^a^	4.14 ± 0.29^ab^

*Bl:* *B.licheniformis* DW4, *Ss:* *S. sesuvii* DW5, *Ps:* *P. suwonensis* DW7 and B mix: *B.licheniformis* DW4 + *S. sesuvii* DW5 + *P. suwonensis* DW7. Value: mean ± S.D, Means shared the same letter(s) are not significantly different, Tuckey test (*p* ≤ 0.05)

The content of total proline varied among treatments as illustrated in Table 6. The B mix treatment displayed the highest proline accumulation at 4.14 ± 0.29 mg/g DW, starkly contrasting the lower values found in the freshwater and wastewater controls.

#### Enzymatic antioxidants assay after 110 days of planting

In Table 7, the results obtained for the relative enzyme activities of catalase, peroxidase, polyphenol oxidase and phenylalanine ammonia lyase in *Vicia faba* leaves, as an effect of different treatments were presented.

**Table 7:** Effect of different treatments with halo-endophytic bacteria; *B.l*,* S.s*,* P.s* and B mix and salt stress of 150 mM NaCl concentration compared to control on catalase activity (CAT), peroxidase activity (POD) and polyphenol oxidase activity (PPO) in *Vicia Faba* and expressed as Unit/g fresh weight

Treatments	CAT (Unit/g)	POX (Unit/g)	PPO (Unit/g)	PAL (Unit/g)
Freshwater control	0.14 ± 0.61^ab^	0.99 ± 0.86^c^	0.86 ± 0.26^d^	0.52 ± 0.09^bc^
Wastewater control	0 ± 0^ab^	2.96 ± 0.69^bc^	1.15 ± 0.12^d^	0.46 ± 0.17^c^
B.l	1.73 ± 1.37^a^	3.88 ± 0.23^b^	2.18 ± 0.76^bc^	0.95 ± 0.12^ab^
S.s	1.26 ± 0.91^ab^	3.86 ± 1.43^b^	2.21 ± 0.08^abc^	0.92 ± 0.19^ab^
P.s	1.27 ± 1.1^ab^	3.5 ± 0.66 ^b^	2.62 ± 0.34^ab^	0.92 ± 0.23^ab^
B mix	1.92 ± 1.47^a^	6.18 ± 1.45^a^	3.03 ± 0.28^a^	1.06 ± 0.26^a^

*Bl:* *B.licheniformis* DW4, *Ss:* *S. sesuvii *DW5, *Ps:* *P. suwonensis* DW7 and B mix: *B.licheniformis* DW4 + *S. sesuvii *DW5 + P. suwonensis DW7. Value: mean ± S.D, Means shared the same letter(s) are not significantly different, Tuckey test (*p* ≤ 0.05)

The results of the catalase test showed a great difference in activity compared with freshwater and wastewater controls as shown in Table 7. The maximum activity was obtained with B mix of 1.92 ± 1.47 Unit/g. fresh weight, while the lowest activity was observed in freshwater control of 0.14 ± 0.61 Unit/g. fresh weight and there is no activity in wastewater control. The activity of peroxidase varied between treatments as illustrated in Table 7. The maximum activity shown in B mix of 6.18 ± 1.45 Unit/g. fresh weight, whereas freshwater and wastewater controls recorded minimum activities.

As shown in Table 7, the highest activity was shown in B mix of 3.03 ± 0.28 Unit/g. fresh weight compared with freshwater and wastewater controls recorded the lowest value. The activity of phenylalanine ammonia lyase varied among treatments as illustrated in Table 7. The maximum activity shown in B mix of 1.06 ± 0.26 Unit/g. fresh weight, whereas freshwater and wastewater controls recorded the minimum activities.

### Soil fertility

Soil fertility analysis was determined as the change of dehydrogenase activity, total bacterial count and total halophytic bacterial count in rhizospheric soil before and after planting (145 days) as shown in Table 8. The dehydrogenase activity, total bacterial count and total halophytic bacterial count elevated in treated samples, particularly those of soil containing B mix and the maximum dehydrogenase activity of 0.896 ± 0.321 and 0.816 ± 0.284 µg TPF/g Dry Soil/min were in soil samples with B mix and *S.s* compared with freshwater and wastewater controls recording the lowest activity (Table 8).

**Table 8:** Measurement of dehydrogenase and total bacterial count in rhizosphere treated with different halo-endophytic bacteria; B.l, S.s, P.s and B mix compared to control, under salt stress 150 mM NaCl concentration before and after planting as an indicator of soil fertility

	Treatments	DHG (µg TPF/g dry soil/min)	CFU/g soil	
			Total bacterial count	Total halophytic bacterial count
Before planting	Before	0.273 ± 0.12^b^	48 × 10^3^ ±2828^d^	0 ± 0 ^f^
After planting	Freshwater control	0.44 ± 0.247^ab^	296 × 10^3^ ±5657^d^	234 × 10^3^ ±5657^d^
	Wastewater control	0.373 ± 0.33^ab^	201 × 10^3^ ±1414^d^	117.5 × 10^3^ ±3536^e^
	***B.l***	0.611 ± 0.036^ab^	335 × 10^3^ ±7071^d^	234 × 10^3^ ±5657^d^
	***S.s***	0.816 ± 0.284^a^	258.5 × 10^5^ ±70,711^c^	460 × 10^3^ ±5657^c^
	***P.s***	0.694 ± 0.194^ab^	141.5 × 10^6^ ±707,107^b^	482.5 × 10^3^ ±3536^b^
	B mix	0.896 ± 0.321^a^	182.25 × 10^6^ ±353,553^a^	503.5 × 10^3^ ±4950^a^

*Bl:* *B.licheniformis *DW4, *Ss:* *S. sesuvii* DW5, *Ps:* *P. suwonensis* DW7 and B mix: B.licheniformis DW4 + *S. sesuvii *DW5 + *P. suwonensis* DW7. Value: mean ± S.D, Means shared the same letter(s) are not significantly different, Tuckey test (*p* ≤ 0.05)

The highest total bacterial count of 182.25 × 10^6^ ±353,553 and 141.5 × 10^6^ ±707,107 CFU/g soil were observed in soil samples with B mix and *P.s* compared with control freshwater and wastewater controls recording the lowest count (Table 8). The highest total halophytic bacterial count of 503.5 × 10^3^ ±4950 and 482.5 × 10^3^ ±3536 CFU/g soil were in soil samples with B mix and *P.s* compared with control freshwater and wastewater controls recording the lowest count (Table 8).

### Impact of faba seeds biopriming on yield parameters under salt stress

After 145 days (Table 9), the maximum number and weight of pods per plant were obtained in treated seeds with B mix at 150 mM NaCl concentration were 1.75 ± 0.5 and 0.58 ± 0.45 g, respectively. The maximum length of pods and number of seeds per pod was obtained in treated seeds with B mix 150 mM NaCl concentration were 4.75 ± 1.06 cm and 2 ± 0, respectively. The maximum weight of seeds per plant and the weight of 100 seeds were obtained in treated seeds with B mix at 150 mM NaCl concentration were 0.56 ± 0.606 g and 33.33 ± 2.8, respectively.

**Table 9:** Different yield parameters of *Vicia Faba* treated with single; *B.l*,* S.s* and *P.s* and a mixture; B mix of halo-endophytic bacteria and cultivated in salt-affected soil after 145 days

Irrigation	Treatments	No pods/plant	Weight of pods/plant	Length of pods	No seeds/pod	Weight of seeds/plant	Weight of 100 seeds
Freshwater	Control	1.33 ± 0.58 ^ab^	0.47 ± 0.16 ^b^	4 ± 0.71 ^b^	1 ± 0.82 ^c^	0.41 ± 0.053 ^b^	23.83 ± 3.75 ^b^
Wastewater	Control	1 ± 0 ^ab^	0.27 ± 0.15 ^cd^	0 ± 2.45 ^d^	1.29 ± 0.95 ^bc^	0.15 ± 0.131 ^de^	11.33 ± 1.15 ^c^
150 mM NaCl conc	***B.l***	1 ± 0 ^ab^	0.31 ± 0.17 ^c^	3.79 ± 1.11 ^c^	1.5 ± 0.58 ^b^	0.26 ± 0.213 ^d^	17.5 ± 2.12 ^bc^
	***S.s***	1.33 ± 0.58 ^ab^	0.37 ± 0.29 ^c^	3.63 ± 0.48 ^c^	1.5 ± 1 ^b^	0.31 ± 0.441 ^c^	18 ± 4.24 ^bc^
	***P.s***	1.6 ± 0.89 ^a^	0.47 ± 0.01 ^b^	4 ± 0.96 ^b^	1.5 ± 0.76 ^b^	0.34 ± 0.03 ^c^	20.38 ± 6.99 ^b^
	B mix	1.75 ± 0.5 ^a^	0.58 ± 0.45 ^a^	4.75 ± 1.06 ^a^	2 ± 0 ^a^	0.56 ± 0.606 ^a^	33.33 ± 2.8 ^a^
Effect of inoculation with halo-endophytic bacteria							
Control		0.8 ^cd^	0.34 ^c^	2.8 ^c^	1.13 ^c^	0.29 ^cd^	11.93 ^d^
B.l		1.13 ^bc^	0.74 ^b^	4.56 ^b^	1.8 ^bc^	0.48 ^bc^	34.26 ^c^
S.s		1.46 ^ab^	0.93 ^ab^	5.23 ^ab^	2.4 ^ab^	0.84 ^a^	38.58 ^c^
P.s		1.53 ^ab^	1.16 ^a^	6.06 ^a^	2.33 ^ab^	0.78 ^ab^	49.94 ^b^
B mix		1.73 ^a^	1.07 ^a^	5.56 ^ab^	2.53 ^a^	0.93 ^a^	73.83 ^a^
Effect of irrigation with different concentration of seawater							
Freshwater		1.47 ^a^	1.35 ^a^	5.5 ^a^	1.95 ^a^	1.07 ^a^	67.73 ^a^
Wastewater		0.80 ^c^	0.24 ^c^	2.40 ^c^	1 ^c^	0.16 ^c^	9.98 ^c^
150 mM NaCl conc		1 ^bc^	0.54 ^bc^	3.57 ^b^	1.42 ^bc^	0.47 ^bc^	41.96 ^b^

### Total protein content in *Vicia Faba* seeds

All treatments showed a positive influence on total protein content compared with freshwater and wastewater controls. The maximum protein content was recorded in B mix of 286.8 ± 3.96 mg/g DW, described in Table 10.

**Table 10:** Effect of different treatments of halo-endophytic bacteria; *B.l*,* S.s*,* P.s* and B mix and salt stress of 150 mM NaCl concentration on total proteins content in *Vicia Faba*

Treatments	Total proteins (mg/g)	
Freshwater control	241.14 ± 1.61^d^	
Wastewater control	216.3 ± 0.98^e^	
B.l	246.08 ± 1.52^cd^	
S.s	253.46 ± 4.89^bc^	
P.s	262.36 ± 3.33^b^	
B mix	286.8 ± 3.96^a^	

*Bl* :*B.licheniformis* DW4, *Ss:* *S. sesuvii *DW5, *Ps:* *P. suwonensis* DW7 and B mix: *B.licheniformis* DW4 + *S. sesuvii *DW5 + *P. suwonensis* DW7. Value: mean ± S.D, Means shared the same letter(s) are not significantly different, Tuckey test (*p* ≤ 0.05)

## Discussion

The semi-field experiment was conducted in the newly-reclaimed Qalabshu soil, which was characterized by high baseline concentrations of EC, Cl^−^, and Na^+^. Due to the extreme salinity sensitivity of *Vicia* and its presence in newly-reclaimed soil is impossible, a method of gradual irrigation was implemented, involving incrementally increasing the salt concentrations in the watering system throughout the growth period [56].

In this study, seeds of *Vicia faba* were bio-primed with PGP bacteria, including the strain *P. suwonensis* DW7 and a bacterial mix (B mix). These treatments effectively increased seed germination percentages under salt stress. The mechanism behind this improvement is attributed to the bacteria’s ability to produce bioactive compounds and stimulate hormonal production, which jointly promote plant growth and disease resistance. This finding is consistent with previous research, including a study by Meza C, et al. [86] on *Phaseolus vulgaris* and a decrease in pre- and post-emergence damping-off and a prior report by Wael D, et al. [62] which noted that these bacteria possess the ability to produce ammonia, siderophores, and digestive enzymes. Ammonia production benefits plants in two ways: it increases soil pH, which suppresses the growth and sporulation of pathogenic fungi, and it directly supplies the essential macronutrient nitrogen. This dual action improves both crop health and biomass by enhancing root and shoot growth [87]. Siderophores indirectly boost plant health by acting as a biological control. These low-molecular-weight compounds sequester iron (Fe^3+^) and other metals from the soil, making them inaccessible to plant pathogens and thus protecting the plant from disease [88] and digestive enzymes that damage fungal pathogens’ cell wall [89].

The initial advantage, observed as increased seed germination and subsequent growth, is attributable to the molecular signaling stimulated by the bio-priming. The production of Phytohormones such as IAA and GA-3 by *P. suwonensis* DW7 and the B mix directly contributes to the maintenance of growth parameters under inhibitory salt conditions. High levels of bacterial IAA are known to modulate root architecture and potentially help maintain turgor and nutrient uptake efficiency under osmotic stress. This hormonal signaling effectively overrides the salt-induced suppression of cell division and expansion, consistent with growth promotion findings in other legumes under stress. While Meza C, et al. [86] observed general growth promotion in *Phaseolus vulgaris*, de Oliveira Lopes ÁL, et al. [90] in lima bean and Mahgoub HA, et al. [56] in *Vicia faba* the magnitude of increase in key yield parameters observed in our *Vicia faba* under severe seawater stress suggests a more efficient stress-adaptation mechanism in *P. suwonensis* DW7.

Visual analyses of the roots of *Vicia faba* revealed that there was no nodulation on the untreated plants (controls), conversely, bacterial treatments showed nodulation. This suggested the ability of these bacteria to fix nitrogen and produce cellulase, which may enhance nodulation. The enzyme likely facilitates bacterial penetration into root hair tissues by softening them, promoting root hair curling, and ultimately leading to nodule development [91]. A symbiotic relationship with the plant through the formed nodules that make atmospheric nitrogen available to the plant in a utilizable organic form as supported by the work of de Oliveira Lopes ÁL, et al. [90].

The root, being in direct contact with the soil, is the most vulnerable to salt stress. Our findings reveal that PGP bio-priming, particularly with *P. suwonensis* DW7 and B mix, induced critical morphological and anatomical adaptations in *Vicia faba* roots, confirming results on common bean and these results were validated by Peña-Valdivia CB, et al. [92], Ramamoorthy Purushothaman RP, et al. [93] and Strock CF, et al. [94] on common bean. Under salt stress, these treatments significantly increased the size of the vascular cylinder and the density of xylem and phloem vessels. This structural enhancement is a vital physiological mechanism for adaptation, hypothesized to maintain water-use efficiency and nutrient transport under high osmotic potential, ensuring water supply to the shoots despite high external salinity. Furthermore, the formation of a thick-walled cell ring beneath the piliferous layer, especially in *P. suwonensis* treatment, is a novel protective barrier that likely restricts Na^+^ or Cl^−^ ion uptake and radial movement, offering superior resistance to salinity compared to the generalized anatomical changes reported in mung bean [70].

One useful indicator of a plant’s health is its photosynthetic pigments like Chl a, Chl b and carotenoids increased in the treatments with bacteria especially *P.s* and B mix compared to controls, this was due to the increase in all parameters of growth of *Vicia faba* such as plant height, root and shoot length and fresh weight and this because Meza C, et al. [86] and de Oliveira Lopes ÁL, et al. [90] have demonstrated that these bacteria can enhance the growth of faba beans under salt stress by producing hormones that promote plant growth, such as GA-3 and IAA.

The improved growth and yield under seawater stress are fundamentally linked to the biochemical defense systems activated by the PGP bacteria. Salinity stress is known to induce severe oxidative stress by generating excessive ROS in plant tissues. We found that the applied bacteria significantly boosted both enzymatic (catalase, peroxidase, polyphenol oxidase, and phenylalanine ammonia lyase) and non-enzymatic antioxidant pathways (total phenolics and total flavonoids). This enhanced capacity for ROS scavenging is a crucial molecular mechanism allowing the *Vicia faba* to mitigate cellular damage. The significant increase in antioxidant enzyme activity observed in our study exceeds the typical baseline responses reported in studies of non-halotolerant PGPB under salt stress [56, 95]. This suggests our strain possesses an exceptional molecular capability for stress defense induction.

In parallel, the bacteria facilitated a marked increase in the accumulation of proline. Proline functions as a multifunctional osmolyte (balancing water potential), a chaperone (protecting enzyme structure), and an additional ROS detoxifier. This osmotic regulatory mechanism is vital for maintaining cell turgor and minimizing water loss under the high external osmotic pressure of saline stress. Crucially, the bacteria’s dual roles of promoting nodule formation and simultaneously elevating proline content contributed significantly to stress tolerance and yield enhancement. Alsenidi MD, et al. [95], Yang J, et al. [96] and Cruz C, et al. [97] suggest a synergistic interplay where improved nitrogen supply directly fuels the biosynthesis of these energy-intensive stress protectants.

Interactions within the rhizosphere are vital for plant growth and soil fertility. Plants provide nutrients to PGPB, which in turn boost the development of plants [98]. This study found that applying bacteria, specifically *P. suwonensis* DW7 and the B mix, significantly increased the total and halophytic bacterial count in the soil after treatment compared to controls. This is likely due to the direct interaction between the plant’s roots and the bacteria applied to the seeds. These findings align with previous research on similar plant-microbe systems by Radhakrishnan S, et al. [99], and Bhise KK, et al. [100].

Since dehydrogenase is an enzyme present only in living microorganisms, its detection in soil signifies active microbial populations. These microbes are key players in breaking down organic matter, a process crucial for plant health. The observed rise in dehydrogenase activity indicates that *P. suwonensis* DW7 and B mix stimulated microbial growth and improved overall soil condition compared to pre-treatment sample and controls and this aligns with [87, 101].

The effects of bacterial isolates on *Vicia faba* yield were assessed through evaluation of yield parameters. The treatments with bacteria increase in all yield parameters compared to controls especially *P. suwonensis* DW7 and B mix. These advantages may be due to the bacteria’s ability to fix nitrogen and secrete chemicals that encourage plant development. Other studies corroborate these findings on *Solanum lycopersicum* [102, 103] and Strawberry [47]. Treatments with *P. suwonensis* DW7 and the B mix consortium led to significant agronomic success, specifically increased yield under severe salinity. This protective effect was mediated by significant anatomical changes in the root (e.g., enlarged vascular cylinder), which subsequently improved the plant’s water status and antioxidant defense capacity.

As a result of PGP capability of the isolates and maximum adaptation to salt stress treatments with bacteria through previous parameters leads to increase in all growth and yield parameters and finally leads to increase in total proteins contents in *Vicia faba* dry seeds in treated seeds with bacteria compared to controls due the ability of these bacteria to make nitrogen fixation and nodules, and this results validated by de Oliveira Lopes ÁL, et al. [90] on lima bean and Cruz C, et al. [97] on maize.

## Conclusions

This study definitively demonstrates the significant potential of halophyte-derived PGPB to overcome severe salinity challenges, particularly for the strategic *Vicia faba* crop in newly-reclaimed soils. Our findings establish that specific PGPB treatments, notably *P. suwonensis* DW7 and the B mix consortium, do not merely buffer stress but actively induce a coordinated defense strategy, resulting in dramatic enhancements in yield and nutritional content. The superior performance is interpreted as a multimodal stress-adaptation mechanism. This mechanism is underpinned by: (1) an immediate physiological response (increased photosynthetic efficiency and antioxidant activity, like CAT); (2) a metabolic adjustment (proline accumulation); and (3) a crucial structural adaptation in the root system (enlarged vascular cylinder and increased xylem/phloem density). Critically, the successful establishment and productivity of inoculated plants in highly saline conditions, where control plants failed, showcase the capacity of these inoculants to raise the effective EC threshold of *Vicia faba* beyond its native tolerance. The validated approach of using novel halophyte PGPB strains under seawater irrigation offers a viable and environmentally friendly solution to unlock agricultural productivity in previously unfeasible saline environments, representing a significant step toward sustainable food security in overpopulated, arid regions like Egypt.

## Supplementary Information

Supplementary Material 1.

## Data Availability

Data is provided within the manuscript.
