# Supplementary material for: Significant impact of Bacillus licheniformis DW4, Salinicoccus sesuvii DW5 and Paenalcaligenes suwonensis DW7, in mitigation of seawater stress on the growth and productivity of Vicia Faba cultivated in Qalabshu semi-field soil
Source: BMC Plant Biol. 2026 Feb 4;26:377. doi: 10.1186/s12870-025-08055-8 (PMC12930873; doi:10.1186/s12870-025-08055-8)
Supplement: Supplementary file 1 — Supplementary Material 1. [file 12870_2025_8055_MOESM1_ESM.docx]

**S1.** Total soluble salts, soluble anions and cations and elements analysis of agricultural wastewater collected from Qalabshu.

| **Analysis** | **Wastewater** |
| --- | --- |
| **Total soluble salts** | |
| pH | 7.24±0.02 |
| EC (dS/m) | 5792±1 |
| EC (ppm) | 6.78±0.01 |
| **Soluble anions (mg.eqv/L)** | |
| CO_3_^-2^ | - |
| HCO _3_^−^ | 1.3±0.02 |
| Cl^−^ | 73.36±0.555 |
| SO₄²⁻ | 12.51±0.01 |
| Total anions | 87.17 |
| **Soluble cations (mg.eqv/L)** | |
| Ca^2+^ | 2.55±0.03 |
| Mg^2+^ | 10.49±0.02 |
| Na^+^ | 72.56±0.04 |
| K^+^ | 1.27±0.02 |
| Total cations | 86.87 |
| Residue sodium carbonate | - |
| Adsorbed sodium (%) | 28.42±0.04 |
| **Elements (mg/L)** | |
| NH_4_^+^ | 3.5±0.01 |
| NO_3_^-^ | 2.1±0.02 |
| B | 0.03±0.001 |
| Cu | 0.01±0.001 |
| Fe | 0.01±0.002 |
| Mn | 0.01±0.001 |
| P | 0.03±0.002 |
| Zn | 0.01±0.001 |
| Value: mean ± S.D. | |

**Note:** EC (Electrical Conductivity), dS/m (deciSiemens per meter), ppm (parts per million), mg.eqv/L (milliequivalents per liter), and all chemical symbols (CO_3_^-2^ (Carbonate), HCO _3_^−^ (Bicarbonate), Cl^−^ (Chloride), SO₄²⁻ (Sulfate), Ca^2+^ (Calcium), Mg^2+^ (Magnesium), Na^+^ (Sodium), K^+^ (Potassium), NH_4_^+^ (Ammonium), NO_3_^-^ (Nitrate), B (Boron), Cu (Copper), Fe (Iron), Mn (Manganese), P (Phosphorus), Zn (Zinc)).
